# Characterization of saliva microbiota’s functional feature based on metagenomic sequencing

**DOI:** 10.1186/s40064-016-3728-6

**Published:** 2016-12-20

**Authors:** Fang Yang, Kang Ning, Xiaowei Zeng, Qian Zhou, Xiaoquan Su, Xiao Yuan

**Affiliations:** 1 Department of Stomatology, Qingdao Municipal Hospital, Qingdao, 266011 Shandong China; 2 Shandong Key Laboratory of Energy Genetics, CAS Key Laboratory of Biofuels and BioEnergy Genome Center, Qingdao Institute of Bioenergy and Bioprocess Technology, Chinese Academy of Sciences, Qingdao, 266101 Shandong China

**Keywords:** Caries, Function, Metagenome, Saliva, Microbiota, Whole-ecosystem sequencing

## Abstract

**Objective:**

Saliva, a mixture of exocrinally secretive fluids, amounts to ~1.5 L daily and harbors numerous microbial inhabitants. However, except the organismal structure of saliva microbiota, the functional profile of saliva microbiota remain elusive.

**Methods:**

Here we used metagenomic sequencing to experimentally reconstruct the global genomic profile of saliva by sequencing total saliva DNA from two healthy and two caries-active (DMFT ≧ 6) adults.

**Results:**

We found that saliva microbiota, representing 30–60% of total saliva DNA in our samples, might carry functional signatures that were site-specific and caries-state-specific. Among microbiota from different hosts, a prominent functional core, but not an organismal core, was identified. Each microbiota exhibited functional redundancy where dominant genomes tend to encode more functional diversity yet without necessarily contributing to dominant functions. Furthermore, genetic polymorphisms of hosts were also unraveled from salivary DNA without apparent physical or sequence bias in human chromosomes.

**Conclusions:**

The microbial functional sensitivity to disease, links to specific functions, and permission of simultaneous genotyping of hosts and microbiota suggested sequencing salivary DNA might be an advantageous venue in uncovering both human and microbial basis of oral infections.

**Electronic supplementary material:**

The online version of this article (doi:10.1186/s40064-016-3728-6) contains supplementary material, which is available to authorized users.

## Background

Human body is the home to numerous symbiotic microbial organisms that with equal number of our own cells (Sender et al. 2016; Sleator 2010). They not just count upon the human body for food and shelter, but also provide the host with metabolic functions far beyond our own physiological capabilities (Sleator 2010). Thus, a “superorganism” view of human body suggested that interpretation on both human and their inhabitant microbiome holds potential for understanding the etiology of human diseases (Baquero and Nombela 2012).

Saliva harbors a complex microbial community (Yang et al. 2012; Zarco et al. 2012). Multiple studies have characterized the organismal structure of saliva microbiota and revealing a high phylogenetic diversity (Keijser et al. 2008; Yang et al. 2012). Moreover, growing evidence from our research groups and others suggested that organismal structure of saliva microbiota is correlated with disease state such as caries (Yang et al. 2012), gingivitis (Huang et al. 2011) and periodontitis (Griffen et al. 2011), thus the organismal structure of saliva microbiota can potentially serve as a proxy to the oral health of the host (Nasidze et al. 2009; Kanasi et al. 2010). However, except the organismal structure of saliva microbiota, the functional profile of saliva microbiota remain elusive and it potential link to health and disease is not well understood (Human Microbiome Project Consortium 2012).

Here we applied metagenomic sequencing on healthy (“H”) and caries-active (DMFT ≧ 6) (“C”) adults, aiming to experimentally reconstruct the global genomic profile of saliva. The objective of this study was firstly to identify the percentage of microbial DNA contained in the saliva DNA samples and to investigate the functional signatures that related with host caries-status. Finally, genetic polymorphisms of hosts were also evaluated from those saliva samples. Although only four samples were generated for such a complex community, with our novel approach in combining sequence technology, combined assembly and various data analysis methods, our study may provide initial snapshot on how to sequencing the total saliva DNA to unravel both human and microbial basis of oral infections.

## Methods

### Study design

All volunteers were given written informed consent in accordance with the sampling protocol with approval of the ethical committee of the Stomatology Hospital, Sun Yat-sen University. They were all unrelated individuals of both genders, aged between 18 and 23 years and shared a relatively homogeneous college-campus living environment in Sun Yat-sen University. To decipher the functional landscape of saliva, four saliva samples (two from the “healthy” individuals as H105, H114 and two from the “caries-active (DMFT ≧ 6)” subjects as C201, C218) were randomly selected for shotgun sequencing of total genomic DNA (Additional file 1: Table S1). Two boys of caries-active (DMFT ≧ 6) patients of DMFT equals to 7 and 9 were taken into this study. Moreover, two healthy individuals of one boy and one girl with DMFT of 0 were included in this study as healthy controls.“Healthy” individuals (DMFT = 0) and “caries-active (DMFT ≧ 6)” subjects (DMFT ≧ 6) were defined based on DMFT index, which measures the number of decayed, missing and filled teeth in epidemiologic surveys of dental caries (Anaise 1984). All reported no antibiotics intake for the preceding at least 6 months and no smoking or tobacco used. All were asked to avoid eating or drinking for 1 h before oral sampling. Those with other oral (for example, periodontitis or halitosis) or systematic diseases were excluded. Subjects were comfortably seated and, after a few minutes of relaxation, they were trained to avoid swallowing saliva and asked to lean forward and spit all the saliva they produced for 5 min into a graduated test tube. Participants were asked to collect at least 5 mL unstimulated saliva in a plastic cup and sample were transferred to a plastic tube and stored at −80 °C. Additional details of “Methods” section can be found in Additional file 2

### Whole-ecosystem sequencing of saliva

For four of the saliva samples (two from the healthy hosts and the other two from caries-active (DMFT ≧ 6) hosts), shotgun pair-end libraries of total saliva genomic DNA were prepared. Each metagenomic DNA libraries was then sequenced on one lane of pair-end 100 or 75 bp flow-cell on Solexa GA-IIx (Illumina, USA). Reads produced were processed via our computational pipelines customized for human oral microbiome analysis (Xie et al. 2010). All sequences were deposited at Sequence Read Archive (http://www.ncbi.nih.gov/sra) under Accession ID SRA049721. The Solexa GA-IIx reads were subjected to quality filtering using FASTX-Toolkit (V0.0.10; http://hannonlab.cshl.edu/) which trimmed low quality read ends: the tailing nucleotides with quality less than 20 were removed. Human sequences were extracted after mapping the reads to human genome sequences using Novoalign (V2.06; http://www.novocraft.com); those paired reads with both mapping quality values exceeding 100 were labeled as host-originating reads and the rest were considered as microbiota-originating reads.

### Functional classification of genes

After removing the human-originating reads from each of the four whole-ecosystem sequencing datasets, over 8.4 million reads were present for each of the healthy saliva microbiota and over 33.1 million reads were for each of the caries-active (DMFT ≧ 6) microbiota. Interestingly, the microbiota-originating reads constitute ~30% of the total number of reads for the two H samples, while they constitute ~50% of that for the two C samples (Additional file 1: Table S2), suggesting the presence of a higher level of microbial DNA in the saliva of caries-active (DMFT ≧ 6) hosts.

None-human short reads were assembled using SOAP (*Short Oligonucleotide Analysis Packag*e) (Li et al. 2009a, b). Information for the raw reads and their assembly was presented in Additional file 1: Table S2. Those contigs longer than 50 bp were submitted to MG-RAST (*The Metagenomics RAST server*) (Glass et al. 2010) for BLASTX against SEED database (http://www.theseed.org/). MG-RAST annotation was performed using an e-value cut-off of 1e-5 to identify abundant genes from the assembled reads (Overbeek et al. 2005; Glass et al. 2010). About 7.1–49.9% of the sequences contain predicted genes with known functions, while 24.6–62.9% of the sequences encode predicted genes with unknown function (Additional file 1: Table S2). Sequences were deposited, and publicly available on the MG-RAST server (H105: 4454806.3; H114-4454815.3; C201: 4454817.3; C218: 4454816.3). The functional assignment based on SEED subsystems was retrieved for four hierarchical levels: subsystem hierarchy 1, subsystem hierarchy 2, subsystem hierarchy 3 and function level. Functional assignment results based on SEED subsystems from additional metagenomic data in MG-RAST server were taken into comparison, including supragingival dental plaques from healthy individuals (MG-RAST IDs: NOCA_01P-4447192.3, NOCA_03P-4447102.3), supragingival dental plaques from caries-active (DMFT ≧ 6) individuals (MG-RAST IDs: CA_04P-4447943.3, CA_06P-4447903.3), cavities plaques (Belda-Ferre et al. 2012) (MG-RAST IDs: CA_06_1.6-4447971.3, CA_05_4.6-4447970.3), human gut microbiota (Turnbaugh et al. 2009a, b) (MG-RAST IDs: TS1-4440452.7, TS2-4440453.6) and cow rumen microbiota (MG-RAST IDs: 640F6-4441679.3, 80F6-4441680.3). Heat maps and PCA (Principle Component Analysis) results of the functional gene composition were generated from MG-RAST using MEV v 2.0 (Saeed et al. 2006) and R (version 2.9.1; http://www.r-project.org/).

MetaGene (Noguchi et al. 2006) was used to predict ORFs from the contigs. The predicted ORFs were aligned to each other using in-house Perl script to construct a non-redundant protein-coding gene set. These non-redundant protein sequences were aligned against the reference databases that include NR and KEGG (Kyoto Encyclopedia of Genes and Genomes; http://www.genome.jp/kegg/) using TBLASTN (e < 10^−5^) and then analyzed by MEGAN (*Metagenome Analyzer*) (Huson et al. 2007). The BLAST comparison thus allowed association of the proteins to their GO-term classification, as well as to a series of KO numbers. The genes and their associated information were thus projected based on their associated KO numbers onto the reference pathways in KEGG using the iPath tool (Letunic et al. 2008).

### Genome and function linkage

Human Microbiome Project (HMP) oral reference genomes were downloaded (http://www.hmpdacc-resources.org/) and concatenated into one large reference sequence. Alignment of the metagenomic reads to the HMP oral reference genomes was conducted using Bowtie (Langmead et al. 2009) to access the abundance of these reference genomes in the four sequenced saliva microbiota (to define the dominant genomes) and the sequence coverage of these reference genomes or their close phylogenetic neighbors in the four microbiota. MG-RAST annotation based on SEED subsystem was retrieved and the extracted nucleotide sequences encoding proteins were searched against the HMP oral reference genomes using BLASTN to identity their originating genomes. The dominant functions in the four levels in SEED and dominant genomes were defined according to their relative abundance. A database recording the diversity, abundance and originating genomes of the genes and their functions was thus constructed based on which the relationship of the dominant function and the dominant genomes would be examined.

### Analysis of human-originating reads

The localization and distribution of Single-Nucleotide Polymorphisms (SNPs) on the four human-host genomes were obtained via the following strategy. *First*, alignments of the human reads to the reference human genome (version hg18) were performed using BWA (*Burrows*-*Wheeler Aligner*) (Li and Durbin 2009). *Second*, variants calling was performed using SAM (Sequence Alignment Map) tools (v 0.1.16) (Li et al. 2009a, b) and GATK (*The Genome Analysis Toolkit*) (v1.0) (McKenna et al. 2010) with default parameters, respectively. *Third*, the intersection of the variants predicted by SAM tools and GATK were taken as the final variant calls. Functional classification of point mutations was performed using ANNOVAR (*functional annotation of genetic variants from high*-*throughput sequencing data*) (Wang et al. 2010). To test the correlation between the identified SNPs and the potential phenotypes of the human hosts, the SNPs were firstly filtered with the criteria of a minimal read depth of 30 (thus more likely to represent functional variations; (Oetting 2011)). Correlations of these filtered SNPs with potential diseases and phenotypes were subsequently identified by searching the Catalog of Published Genome-Wide Association Studies (http://www.genome.gov/).

## Results

### Functional features of healthy and caries-active (DMFT ≧ 6) saliva microbiota

**Fig. 1:**
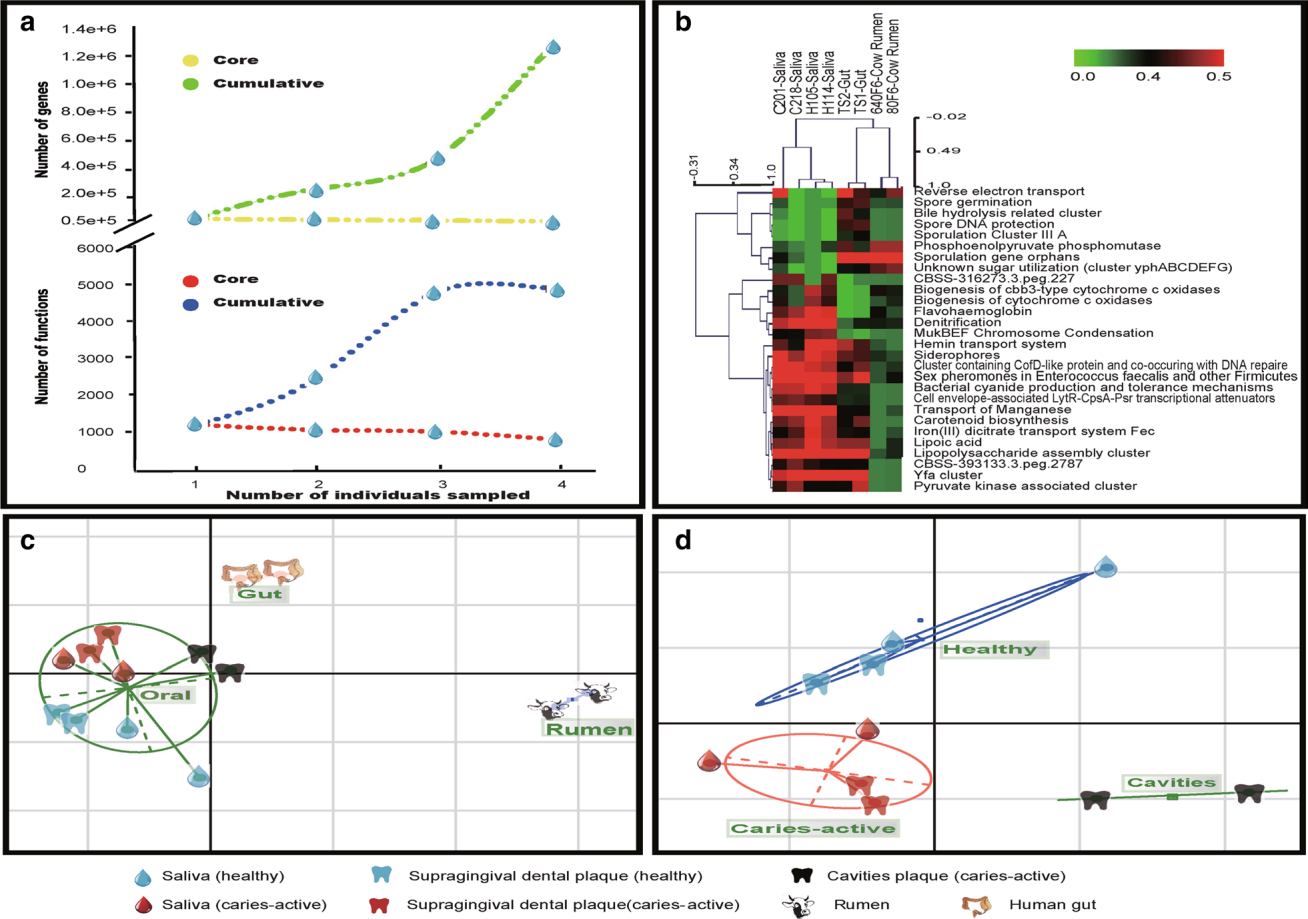
Functional, ecological and epidemiological features of saliva microbiota. **a** The conservation and divergence of gene sequences and encoded functions among the four sequenced human saliva microbiota. **b** Functional signatures of human saliva microbiota. Blocks of gene categories relatively enriched (in *red*) or depleted (in *green*) in each microbiota were highlighted. Comparison of the microbiota was based on functional annotations on MG-RAST Subsystem Level 2. **c** Functional clustering of microbiota from different mammals or body sites. They include human mouth [including saliva, supragingival dental plaques (Belda-Ferre et al. 2012) and cavities plaques (Belda-Ferre et al. 2012)], human gut (Turnbaugh et al. 2009a, b) and cow rumen (originated from MG-RAST server). Results were generated by Principal Component Analysis (PCA). **d** Functional clustering of oral microbiota from different oral sites or host states. Those from saliva [both healthy and caries-active (DMFT ≧ 6)], supragingival dental plaques [both healthy and caries-active (DMFT ≧ 6)] and cavities plaques were shown

Four saliva samples (two from healthy hosts and two from caries-active (DMFT ≧ 6) hosts; Additional file 1: Table S1) were selected for whole-ecosystem-sequencing using total genomic DNA. In each of the four datasets, a significant portion of reads (30% for H samples and 50–60% for C samples) was found originated from microbes (Additional file 1: Table S2). The annotations by MG-RAST based on SEED subsystems (Additional file 1: Table S3) consist of four levels: Subsystem hierarchy 1, 2, 3 and “function”. In hierarchy 1, the predominant functional categories included Proteins Metabolism (~6.0%), Carbohydrate Metabolism (~9.4%), Amino Acids and Derivatives (~6.8%), Cofactors, Vitamins, Prothetic groups and Pigments (~6.0%); DNA Metabolism (~6.0%), RNA Metabolism (~5.4%). The four saliva microbiota encoded totally 5854 recognizable functions, derived from 509,644 gene sequences (Fig. 1a). However, a core (shared by each microbiota) of 2203 functions derived from 33,487 gene sequences was identified (Fig. 1a). At each of the three Subsystem hierarchies, saliva microbiota from different hosts exhibited a high degree of functional similarity (Fig. 1b, Additional file 1: Table S3), a finding also supported by comparison based on Gene Ontology (Additional file 1: Table S4). Thus the high degree of divergence in taxonomy (OTU; Yang et al. 2012) contrasts sharply with the relative functional conservation among the four saliva microbiota. However, reconstructed metabolic maps of the four saliva microbiota revealed that the two H microbiota harbored higher levels of unique proteins in the pathways of ‘Glycolysis/gluconeogenesis’ (~40.5% enriched), ‘Pyruvate metabolism’ (~26.9% enriched) and ‘Pentose phosphate metabolism’ (~16.9% enriched) than the two C microbiota (Additional file 3: Figure S1A, B, C and D). Thus saliva microbiota of each human host represents an ‘island’ inhabited by protein collections that encode globally similar functions yet with difference at specific metabolic pathways and with even higher divergence at their residential organisms.

### Ecological features of human saliva microbiota

Human saliva microbiota carried site-specific functional signatures (Fig. 1b), revealed via comparisons with human gut (Turnbaugh et al. 2009a, b) (MG-RAST IDs: TS1-4440452.7 and TS2-4440453.6) and cow rumen microbiota (MG-RAST IDs: 640F6-4441679.3 and 80F6-4441680.3). For example, in human saliva (relative to human gut), subsystems such as ‘Bile hydrolysis related cluster’, ‘Spore DNA protection’, ‘Spore germination’, ‘Sporulation Cluster III A’, ‘Sporulation gene orphans’ and ‘Reverse electron transport’ were depleted, while ‘Denitrification’, ‘Flavohaemoglobin’, ‘Biogenesis of cbb3-type cytochrome c oxidases’, ‘Biogenesis of cytochrome c oxidases’ and ‘MukBEF Chromosome Condensation’ were enriched. Compared with rumen, human saliva was over-represented by ‘Bacterial cyanide production and tolerance mechanisms’, ‘Carotenoid biosynthesis’, ‘Denitrification’, ‘Flavohaemoglobin’, ‘MukBEF Chromosome Condensation’, while under-represented in ‘Phosphoenolpyruvate phosphomutase’ and ‘Reverse electron transport’.

Besides carrying site-specific signatures, functional profiles of saliva microbiota may potentialy be indicative of host health state. When microbiota of supragingival dental plaques from healthy or caries-active (DMFT ≧ 6) individuals and plaque microbiota from caries cavities were included in the functional comparison, the oral microbiota (including both saliva and plaques) were not only separated from the human gut and rumen ones (Fig. 1c), but also could be clustered according to host health status (Fig. 1d).

### Links between genomes and functions in human saliva microbiota

**Fig. 2:**
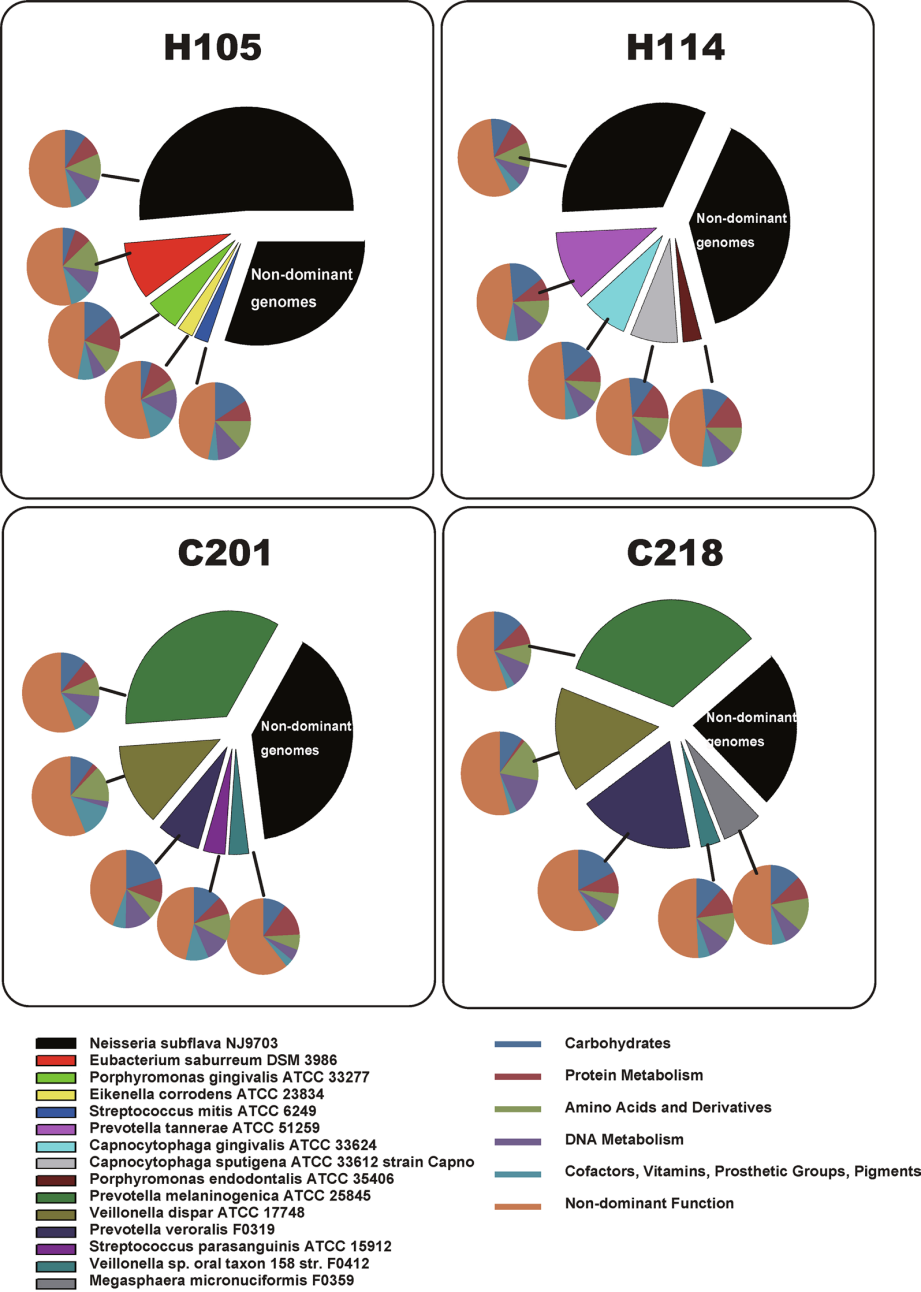
Dominant genomes and their encoded functions in each of the four human saliva microbiota. For a specific organismal lineage (i.e. a genome or a set of genomes from a phylogenetic clade), the encoded dominant functions were similar across the samples. However, different organismal lineages in saliva could encode distinct functions

**Fig. 3:**
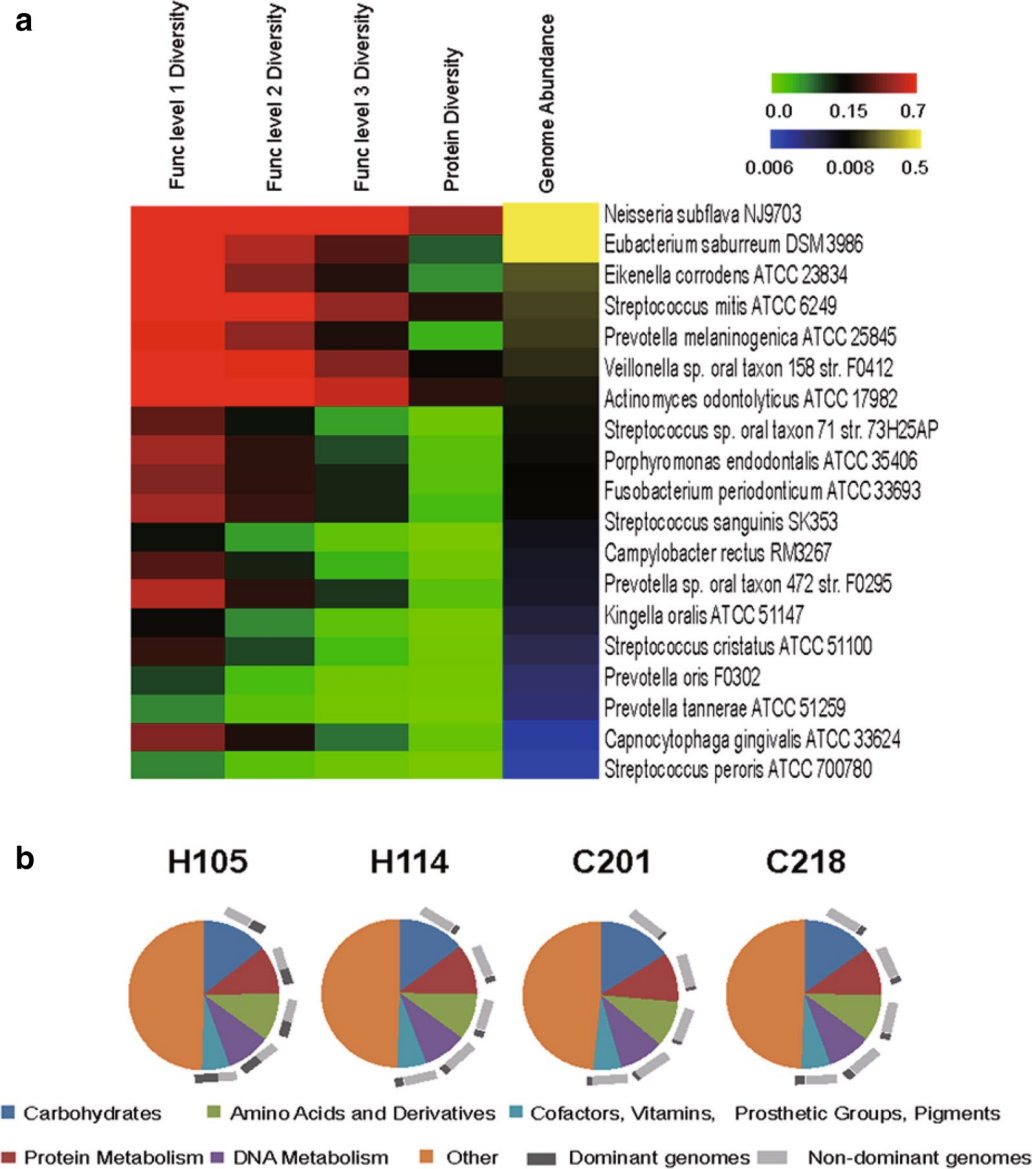
Links between the residential genomes and the encoded functions in human saliva microbiota. **a** Dominant genomes and their encoded functions for sample of H105 (additional microbiota were shown in Additional file 5: Figure S3). Dominant genomes encode the most diverse functions. *Upper color bar* represents fraction of functions encoded by a particular genome among the total number of functions encoded by the microbiota. *Lower color bar* shows the relative abundance of genomes in a microbiota, i.e. the fractions of reads mapped to a genome among all mapped reads. **b** Dominant functions and their originating genomes in each of the four saliva microbiota. Across the four saliva microbiota, the dominant functions and their relative representations were conserved; however, the majority of them were not mainly contributed by dominant genomes

To probe the organism-function relationship in saliva microbiota, a database recording the diversity, abundance and originating genomes of the proteins and their functions in saliva microbiomes was built. We found that, *First*, for the most dominant genomes (those genomes with the top five most abundant hits; Fig. 2 and Additional file 4: Figure S2), the two H microbiota featured *Neisseria subflava* NJ9703, while the two C microbiota featured *Prevotella melaninogenica* ATCC 25845. For a specific organismal lineage (i.e. a genome or a set of genomes from a phylogenetic clade), the encoded dominant functions were similar across different samples. However, different organismal lineages could encode distinct functions. For example, *Prevotella veroralis* F0319, *Prevotella tannerae* ATCC 51259 and *Streptococcus mitis* ATCC 6249 encoded significantly more carbohydrates metabolism genes (16–20%), while *Eubacterium saburreum* DSM 3986 and *Eikenella corrodens* ATCC 23834 encoded significantly fewer of them (5–7.52%) (Fig. 2). *Second*, at each level of the functional hierarchy, with the decreasing abundance of the dominant genomes, diversity of the encoded functions showed a gradually decreasing trend (Fig. 3a, Additional file 5: Figure S3). Thus the dominant genomes encoded more diverse functions. *Thirdly,* the dominant functions, at each level of the hierarchy, were similar across the four microbiota (Fig. 3b), suggesting functional gene structures were more conservative among hosts than organismal structures.

Interestingly, the dominant functions [those functions with the top five most abundant hits: ‘Amino Acids and Derivatives’, ‘Carbohydrates’, ‘DNA Metabolism’, ‘Protein Metabolism’ and ‘(Cofactors, Vitamins, Prosthetic Groups, Pigments)’] were not mainly contributed by dominant genomes. In H105 and H114, dominant genomes (totally accounting for 70.0% in H105 and 61.1% in H114 in relative abundance) respectively contributed only 34.1–56.8 and 19.1–27.7% of the sequences in the various functional categories; while in C201 and C218, dominant genomes (totally accounting for 60.0% in C201 and 76.3% in C218) respectively contributed merely 5.9–10.9 and 12.7–23.8% (Fig. 3b). In fact, dominant functions tend to originate from a more diverse set of genomes (Additional file 6: Figure S4). Therefore, dominant genomes tended to display more functional diversity, while their contributions to dominant functions were not as apparent.

### Host genotype revealed via whole-ecosystem sequencing of saliva

In each of the four saliva whole-ecosystem-sequencing datasets, 70% of the total reads in H samples and 40–50% in C samples originated from host genomes (Additional file 1: Table S2). No apparent sequence or physical bias was detected in their distribution on the reference human genome (Additional file 7: Figure S5A): all host-derived short reads are distributed on the somatic chromosomes with similar density (Additional file 7: Figure S5B), demonstrating the value of saliva DNA for genome-wide analysis of the genetic variations in human hosts. In each dataset, despite a relatively low average sequence-coverage of the human genome (2.68 for H105, 3.08 for H114, 2.81 for C201 and 2.74 for C218), 107,370–635,676 candidate SNPs were identified, representing 25–30% of the ~1.8 million total SNPs (Sherry et al. 2001) in a human genome. The SNPs were distributed in each of the somatic chromosomes with similar density in each of the four datasets (Additional file 8: Figure S6 and Additional file 1: Table S5). In each dataset, ~36% of SNPs were located in intronic regions, over 50% in intergenic regions and 1.1–1.4% in exonic regions (Additional file 7: Figure S5C and Additional file 1: Table S5).

In each of the four metagenomic datasets, 1071–4329 human genes were found with SNPs. Most of such genes were house-keeping genes. In H114, two of the “SNPs with high read-depth” (>30X sequence coverage; Additional file 1: Table S6; (Oetting 2011)), [chr17: 41400462] and [chr17:41400511], resulted in two non-synonymous mutations in *MAPT* (microtubule-associated protein tau), which was associated with inheritable Parkinson’s disease (Trotta et al. 2011). This genotype was subsequently found consistent with the host’s family history. Therefore, in addition to microbial genotypes, saliva can provide readily and valuable access to host genotypes (Quinque et al. 2006).

Here we used metagenomic sequencing to experimentally reconstruct the global genomic profile of saliva by sequencing total saliva DNA from two healthy (“H”) and two caries-active (DMFT ≧ 6) (“C”) adults. We found that saliva microbiota, representing 30–60% of total saliva DNA in our samples, may carry functional signatures that were site-specific and caries-state-specific. Among microbiota from different hosts, a prominent functional core, but not an organismal core, was identified. Furthermore, genetic polymorphisms of hosts were also unraveled from those saliva samples without apparent physical or sequence bias in human chromosomes. Thus sequencing of total saliva DNA might be an advantageous venue in uncovering both human and microbial basis of oral infections.

## Discussion

Pioneering metagenomic studies in the human microbiome are mainly focused in the gut ecosystem field (Gill et al. 2006). Recently, despite numerous studies analyzing species richness in oral microbiomes through sequencing of PCR-amplified rRNA genes (Griffen et al. 2011; Huang et al. 2011; Yang et al. 2012), the true genomic composition of the oral microbiota remains elusive. Xie et al. (2010) was the first reporter of the oral metagenome based on one plaque sample, followed by Belda-Ferre’s et al. (2012) study on six supragingival plaque samples from different caries status. However, for saliva microbiota, there’s only one report on a single sample from a healthy individual (Lazarevic et al. 2012), yet what they focused is the bacterial profile, instead of functional feature. In this study, we choose unstimulated saliva as the origin of saliva samples, to avoid the potential that procedure of stimulated saliva sample collective may dilute or lead to quantity difference of the saliva microbiota (Schafer et al. 2014; Yakob et al. 2014; Belstrom et al. 2016). Here we report the first metagenomic analysis of saliva using WGS sequencing to experimentally reconstruct the global genomic profile of saliva from two healthy (“H”) and two caries-active (DMFT ≧ 6) (“C”) adults.

Under the current notion, salivary functions mainly include (de Almeida Pdel et al. 2008): (i) lubrication and protection of oral tissues, acting as a barrier against irritants; (ii) buffering, cleaning and anti-bacterial activities; (iii) maintenance of tooth integrity; (iv) taste and digestion. However, as were observed from these four saliva samples, microbial DNA can represent 30–60% of total saliva DNA (the remaining 40–70% was human-originated), suggesting the need to consider microbial activities as additional contributors to normal salivary function. For example, the enrichment of denitrification-gene in saliva microbiota (mainly in *Neisseria* and *Actinomyces*) provided a potential biogenesis mechanism for the observed NO_2_^−^ and NO in human mouth (Duncan et al. 1995). Dietary NO_3_^−^ is concentrated in salivary glands after being absorbed from intestine into blood, resulting in millimolar concentration of salivary NO_3_^−^ (Lundberg et al. 2008). Conversion of oral NO_3_^−^ to NO_2_^−^ or NO is implicated in periodontal and caries diseases, and moreover, regulates signaling in human immune system, stimulates gastric blood flow, and regulates vasodilatation (Brennan et al. 2003; Lundberg et al. 2008). On the other hand, NO exhibits microbial cytotoxicity by facilitating nitrosative stress and inhibiting heme-containing proteins within the aerobic respiratory chain (Forrester and Foster 2012). Interestingly, flavohaemoglobin (flavoHb), another function specifically enriched in saliva microbiota (mainly *Actinomyces* and *Gemella*), converts NO to inert nitrate (NO_3_^−^) and undergoes catalytic NO regeneration via flavin-dependent reduction (Forrester and Foster 2012). These predicted microbial functions in saliva will need to be experimentally validated in in vitro or in vivo models.

Furthermore, our data suggested that the functional profiles of saliva microbiota may potentially be indicative of host health state, as they were clustered according to the caries state of the hosts. Our result suggested that (i) functional profiles of saliva microbiota can be sensitive to the caries state; and (ii) changes in host health state can be more important than difference in ecological sites in determining the functional profiles of oral microbiota. The roots of such sensitivity in microbial function to host disease/health state can be traced to the genome-function links in saliva microbiota. We have previously demonstrated a high degree of divergence in organismal structure and a minimal organismal core in human saliva microbiota among host individuals (Yang et al. 2012), however it was not clear how the collective functions of saliva microbes were assembled from the component organisms. Based on our result, conservation of human saliva microbiomes is higher at the functional level than at the organismal level. Each genome tends to encode specific functions, thus the abundance-change of community structure can result in functional alteration of the community. However, dominant genomes tend to encode more diverse functions, while their contributions to dominant functions are not apparent. A set of core functions encoded in multiple abundant organismal lineages with a high degree of redundancy is present, thus not a single phylotype is irreplaceable. Each individual microbiota can thus be viewed as an ‘island’ inhabited by unique collections of microbial phylotypes: different species assemblages converge on shared core functions provided by distinct organismal components. These findings explained the absence of a microbial organismal core among hosts (Yang et al. 2012), as well as the presence of a large functional core characteristic of the disease state of caries.

In addition to microbial genotypes, saliva can provide readily and valuable access to host genotypes. Among the 1485–7341 exonic SNPs in each dataset, 44–47% corresponded to non-synonymous changes. Distributions of SNPs derived from our shallow sequencing of total saliva DNA were actually similar to those from human-genome deep-sequencing (where 0.7–0.9% SNPs are exonic and 48–52% of the exonic SNPs are non-synonymous (Xue et al. 2010; Le and Durbin 2011), with the differences between the two strategies as low as from 0.5% for exonic regions (*t* test *p* value = 0.005) to ~5% for non-synonymous coding-sequence changes (*p* value = 0.020). In H114, two non-synonymous mutations in *MAPT* (microtubule-associated protein tau), which was associated with inheritable Parkinson’s disease (Trotta et al. 2011) were identified. This genotype was subsequently found consistent with the host’s family history. Therefore, in addition to microbial genotypes, saliva can provide readily and valuable access to host genotypes (Quinque et al. 2006). These results demonstrated that the representation of human genomic DNA in saliva is largely complete and unbiased and that human genetic polymorphisms such as SNPs can be unraveled via saliva whole-ecosystem-sequencing without apparent physical or sequence bias.

The microbial sensitivity to host disease state, links to systematic body functions, easy accessibility and non-invasiveness in sampling, and permission of simultaneous genotyping of hosts and microbiota suggested sequencing of total saliva DNA might be an advantageous venue in uncovering both human and microbial basis of oral infections.

This study is a pilot short-gun metagenomic sequencing and data analysis of the human saliva microbiome. The primary purpose of this pilot study was to describe the genomic composition of the saliva, showing a number of technical and practical issues in metagenomic sequencing and data analysis, like the percentage of human-originated or microbiome-originated content in salivary DNA, what software or database should be used for functional assignment or SNP analysis, the link between the members and function among the community and indication of microbial activities’ contribution to host’s normal or diseased state etc. It is our hope that this information will prove useful for other investigators in the oral microbiome research community. The results obtained are interesting but are secondary to the technical issues resolved during this pilot study. We expect that in the future, upon availability of funding, more biology-oriented and large-sample based study could be applied to unravel previously unappreciated microbe-mediated functions in salivary, as well as new strategies for manipulating the role of saliva in health and diseases.

## Additional files

**Additional file 1.** Supplementary table.

**Additional file 2.** SOM-supplementary online material

**Additional file 3: Figure S1.** Global metabolic maps of the four human saliva microbiota. The metabolic profiles were constructed by projecting the non-redundant proteins encoded in the microbiota to the reference metabolic pathways in KEGG.

**Additional file 4: Figure S2.** Relative abundance of the oral reference genomes in HMP (Human Microbiome Project) in each of the four sequenced human saliva microbiota. The top fifteen genomes with the most abundant hits are shown, in an order based on their average abundance in the four microbiota.

**Additional file 5: Figure S3.** Dominant genomes and their encoded functions in the saliva microbiota of H114, C201 and C218. The color bar shows the relative abundance of genomes or fraction of functions encoded by a particular genome in a microbiota.

**Additional file 6: Figure S4.** Diversity of the genomes that contribute the dominant functions. Dominant functions tend to originate from more diverse genomes. The upper color bar represents the diversity of the originating genomes for each functional category. The lower color bar shows the relative abundance of the functional categories.

**Additional file 7: Figure S5.** Whole-ecosystem sequencing of saliva reveal genetic polymorphisms of the hosts. (A) Genome-wide distribution patterns of human-originating reads in the four saliva metagenomes. (**B**) Correlation between sizes of each of the 22 somatic chromosomes and the number of human-originating reads mapped to that chromosome. (**C**) Physical locations and distribution of SNPs on the host genomes.

**Additional file 8: Figure S6.** Genome-wide distribution of human SNPs in each of the four saliva whole-ecosystem-sequencing datasets.
