# Supplementary material for: Characterization of saliva microbiota’s functional feature based on metagenomic sequencing
Source: Springerplus. 2016 Dec 20;5(1):2098. doi: 10.1186/s40064-016-3728-6 (PMC5174016; doi:10.1186/s40064-016-3728-6)
Supplement: Supplementary file 1 — Additional file 1. Supplementary table. [file 40064_2016_3728_MOESM1_ESM.docx]

**Table S1. The human subjects sampled for saliva genomic DNA in this study.**

| **Sample ID** | **Group** | **DMFT index** | **Gender** | **Age** |
| --- | --- | --- | --- | --- |
|  |  |  |  |  |
| H105 | Healthy | 0 | Female | 18 |
| H114 | Healthy | 0 | Male | 20 |
| C201 | High-caries | 7 | Male | 18 |
| C218 | High-caries | 9 | Male | 18 |

**Table S2. Summary of the four human saliva whole-ecosystem sequencing datasets.**

|  | **Sample ID** | **H105** | **H114** | **C201** | **C218** |
| --- | --- | --- | --- | --- | --- |
| Data info | Sequencing info | PE, 2*76 | PE, 2*100 | PE, 2*100 | PE, 2*100 |
|  | Num. of Solexa reads | 28,755,494 | 62,136,130 | 62,339,830 | 63,922,178 |
|  | Total bases (Gb) | 0.6 | 1.9 | 3.3 | 3.5 |
| Human Reads | Human reads | 20,287,686  (70.6%) | 42,950,170  (69.1%) | 29,205,318  (47.8%) | 28,360,916  (44.4%) |
|  | Total bases (Kb) | 1,196,973 | 3,951,415 | 2,686,889 | 2,297,234 |
|  | Genome coverage | 0.4 | 1.32 | 0.9 | 0.77 |
|  | Num. of SNPs | 104,976 | 621,947 | 501,619 | 297,373 |
|  | Average SNP coverage | 2.68 | 3.08 | 2.81 | 2.74 |
|  | Num. of genes with SNP | 1,071 | 4,329 | 3,582 | 2,191 |
| Non-human Reads  (microbiota -  originating) | Non-human reads  (microbiome-originating) | 8,468,493  (29.4%) | 19,185,960  (30.9%) | 33,134,512  (53.2%) | 35,561,262  (55.6%) |
|  | Num. of contigs | 704,165 | 8,897,370 | 11,464,526 | 2,614,746 |
|  | Num. of contigs (>50bp) | 137,415 | 4,730,625 | 5,759,943 | 739,889 |
|  | Mean contig length (bp) | 109±121 | 84±28 | 90±67 | 127±175 |
|  | Mean GC percent (%) | 48±9 | 44±10 | 44±11 | 43±9 |
|  | Predicted proteins with known functions | 68,533  (49.9%) | 336,212  (7.1%) | 687,081  (11.9%) | 343,056  (46.4%) |
|  | Predicted proteins with unknown function | 33,794  (24.6%) | 2,976,892  (62.9%) | 3,371,980  (58.5%) | 189,382  (25.6%) |

**Table S3. Functional patterns encoded in the four human saliva whole-ecosystem sequencing datasets.** The functional patterns of H105 (MG-RAST ID: 4454806.3), H114 (MG-RAST ID: 4454815.3), C201 (MG-RAST ID: 4454817.3), C218 (MG-RAST ID: 4454816.3) were identified via MG-RAST and were characterized by relative abundance of functional categories (only the top 20 shown) at Subsystem hierarchy levels 1, 2, 3 and function level.

|  | **Categories** | **H105** | **H114** | **C201** | **C218** |
| --- | --- | --- | --- | --- | --- |
| Level 1 | *Clustering-based subsystems* | 16.59% | 16.60% | 16.35% | 17.30% |
|  | *Protein Metabolism* | 9.43% | 10.63% | 9.35% | 10.14% |
|  | *Carbohydrates* | 9.42% | 9.11% | 9.98% | 9.21% |
|  | *Miscellaneous* | 8.66% | 8.11% | 8.15% | 8.24% |
|  | *Amino Acids and Derivatives* | 6.96% | 6.64% | 7.15% | 6.44% |
|  | *Cofactors, Vitamins, Prosthetic Groups, Pigments* | 6.08% | 6.08% | 5.86% | 5.94% |
|  | *DNA Metabolism* | 5.71% | 5.85% | 5.79% | 6.50% |
|  | *RNA Metabolism* | 5.50% | 5.57% | 4.99% | 5.32% |
|  | *Cell Wall and Capsule* | 4.77% | 4.73% | 4.47% | 4.64% |
|  | *Nucleosides and Nucleotides* | 3.49% | 3.89% | 3.66% | 3.81% |
|  | *Membrane Transport* | 3.36% | 3.48% | 3.77% | 3.92% |
|  | *Virulence, Disease and Defense* | 2.25% | 2.17% | 2.75% | 2.42% |
|  | *Stress Response* | 2.42% | 2.40% | 2.35% | 2.16% |
|  | *Respiration* | 2.62% | 2.56% | 2.25% | 1.89% |
|  | *Fatty Acids, Lipids, and Isoprenoids* | 2.63% | 2.24% | 2.22% | 2.12% |
|  | *Phages, Prophages, Transposable elements,Plasmids* | 1.75% | 1.90% | 1.93% | 1.98% |
|  | *Cell Division and Cell Cycle* | 1.89% | 1.89% | 1.78% | 1.97% |
|  | *Regulation and Cell signaling* | 1.64% | 1.52% | 1.69% | 1.52% |
|  | *Iron acquisition and metabolism* | 1.18% | 1.00% | 0.92% | 0.98% |
|  | *Nitrogen Metabolism* | 0.90% | 0.85% | 0.84% | 0.72% |
| Level 2 | *Plant-Prokaryote DOE project* | 6.91% | 6.46% | 6.55% | 6.59% |
|  | *Protein biosynthesis* | 5.71% | 6.38% | 5.54% | 6.25% |
|  | *RNA processing and modification* | 3.57% | 3.52% | 3.25% | 3.46% |
|  | *Central carbohydrate metabolism* | 3.31% | 3.26% | 3.08% | 2.85% |
|  | *Folate and pterines* | 2.92% | 2.84% | 2.73% | 2.55% |
|  | *DNA repair* | 2.41% | 2.38% | 2.39% | 2.77% |
|  | *DNA replication* | 2.39% | 2.42% | 2.30% | 2.60% |
|  | *Lysine, threonine, methionine, and cysteine* | 2.12% | 2.04% | 2.19% | 2.13% |
|  | *Purines* | 1.64% | 1.70% | 1.57% | 1.65% |
|  | *Sugar utilization in Thermotogales* | 1.47% | 1.37% | 1.56% | 1.53% |
|  | *Resistance to antibiotics and toxic compounds* | 1.39% | 1.36% | 1.60% | 1.47% |
|  | *Di- and oligosaccharides* | 1.37% | 1.21% | 1.48% | 1.38% |
|  | *Capsular and extracellular polysaccharides* | 1.26% | 1.30% | 1.41% | 1.44% |
|  | *Protein degradation* | 1.16% | 1.19% | 1.08% | 1.02% |
|  | *One-carbon Metabolism* | 1.15% | 1.39% | 1.29% | 1.26% |
|  | *Transcription* | 1.12% | 1.27% | 1.05% | 1.11% |
|  | *Peptidoglycan Biosynthesis* | 1.12% | 1.19% | 1.11% | 1.25% |
|  | *Gram-Negative cell wall components* | 1.09% | 1.10% | 0.90% | 0.80% |
|  | *Phages, Prophages* | 1.08% | 1.08% | 1.23% | 1.16% |
|  | *Electron donating reactions* | 1.07% | 1.01% | 0.79% | 0.73% |
| Level 3 | *DNA-replication* | 1.44% | 1.39% | 1.35% | 1.52% |
|  | *YgfZ* | 1.36% | 1.35% | 1.29% | 1.19% |
|  | *tRNA modification Bacteria* | 1.13% | 1.25% | 1.14% | 1.24% |
|  | *Universal GTPases* | 1.01% | 1.19% | 1.04% | 1.13% |
|  | *Serine-glyoxylate cycle* | 0.97% | 1.17% | 1.09% | 1.08% |
|  | *5-FCL-like protein* | 0.90% | 0.75% | 0.69% | 0.65% |
|  | *Methionine Biosynthesis* | 0.70% | 0.66% | 0.74% | 0.74% |
|  | *PROSC* | 0.79% | 0.70% | 0.69% | 0.65% |
|  | *Glycolysis and Gluconeogenesis* | 0.68% | 0.73% | 0.64% | 0.75% |
|  | *De Novo Purine Biosynthesis* | 0.76% | 0.69% | 0.58% | 0.62% |
|  | *Purine conversions* | 0.59% | 0.70% | 0.66% | 0.67% |
|  | *COG1399* | 0.69% | 0.57% | 0.58% | 0.63% |
|  | *Iojap* | 0.53% | 0.60% | 0.58% | 0.57% |
|  | *Glycerolipid and Glycerophospholipid Metabolism in Bacteria* | 0.68% | 0.48% | 0.54% | 0.52% |
|  | *Heat shock dnaK gene cluster extended* | 0.47% | 0.58% | 0.55% | 0.61% |
|  | *DNA repair, bacterial* | 0.49% | 0.50% | 0.57% | 0.64% |
|  | *Ribosome LSU bacterial* | 0.56% | 0.55% | 0.47% | 0.61% |
|  | *Glycolysis and Gluconeogenesis, including Archaeal enzymes* | 0.49% | 0.56% | 0.47% | 0.57% |
|  | *Proteolysis in bacteria, ATP-dependent* | 0.46% | 0.57% | 0.49% | 0.48% |
|  | *RNA methylation* | 0.51% | 0.48% | 0.48% | 0.53% |
| Function | *Decarboxylase* | 0.32% | 0.36% | 0.37% | 0.41% |
|  | *GTP-binding protein* | 0.31% | 0.37% | 0.35% | 0.36% |
|  | *DNA polymerase III alpha subunit (EC 2.7.7.7)* | 0.18% | 0.22% | 0.27% | 0.30% |
|  | *DNA-directed RNA polymerase beta&#39; subunit (EC 2.7.7.6)* | 0.21% | 0.24% | 0.18% | 0.22% |
|  | *DNA-directed RNA polymerase beta subunit (EC 2.7.7.6)* | 0.21% | 0.23% | 0.18% | 0.20% |
|  | *TonB-dependent receptor* | 0.15% | 0.16% | 0.16% | 0.29% |
|  | *Excinuclease ABC subunit A* | 0.14% | 0.19% | 0.18% | 0.20% |
|  | *Protein export cytoplasm protein SecA ATPase RNA helicase (TC 3.A.5.1.1)* | 0.15% | 0.16% | 0.15% | 0.17% |
|  | *Translation elongation factor G* | 0.13% | 0.16% | 0.14% | 0.17% |
|  | *Phosphoribosylformylglycinamidine synthase, synthetase subunit (EC 6.3.5.3)* | 0.18% | 0.15% | 0.11% | 0.14% |
|  | *GTP pyrophosphokinase* | 0.17% | 0.13% | 0.12% | 0.15% |
|  | *DNA gyrase subunit A (EC 5.99.1.3)* | 0.13% | 0.14% | 0.14% | 0.15% |
|  | *GTP pyrophosphokinase (EC 2.7.6.5)* | 0.16% | 0.13% | 0.11% | 0.14% |
|  | *LSU rRNA* | 0.14% | 0.12% | 0.14% | 0.15% |
|  | *Leucyl-tRNA synthetase (EC 6.1.1.4)* | 0.12% | 0.16% | 0.12% | 0.15% |
|  | *Carbamoyl-phosphate synthase large chain (EC 6.3.5.5)* | 0.12% | 0.15% | 0.14% | 0.12% |
|  | *Isoleucyl-tRNA synthetase (EC 6.1.1.5)* | 0.12% | 0.16% | 0.12% | 0.14% |
|  | *Phosphoribosylformylglycinamidine synthase, glutamine amidotransferase subunit (EC 6.3.5.3)* | 0.17% | 0.14% | 0.10% | 0.13% |
|  | *Valyl-tRNA synthetase (EC 6.1.1.9)* | 0.13% | 0.15% | 0.12% | 0.13% |
|  | *GTP pyrophosphokinase (EC 2.7.6.5), (p)ppGpp synthetase I* | 0.16% | 0.12% | 0.10% | 0.14% |

**Table S4. Functional patterns for each of the four human saliva microbiota as defined by Gene Onotology terms.** Colors represent the level of abundance for each GO term.

| **H105** | **H114** | **C201** | **C218** | **GO Term** | **GO ID** |
| --- | --- | --- | --- | --- | --- |
| 22,044 | 21,916 | 22,485 | 23,738 | Molecular_function | GO:0003674 |
| 7,749 | 7,625 | 7,098 | 7,597 | Cytoplasm | GO:0005737 |
| 7,966 | 7,625 | 7,098 | 7,597 | Cell | GO:0005623 |
| 6,583 | 6,278 | 6,931 | 6,510 | Metabolic process | GO:0008152 |
| 4,674 | 4,041 | 4,173 | 4,126 | Catalytic activity | GO:0003824 |
| 3,244 | 3,696 | 3,136 | 3,780 | Translation | GO:0006412 |
| 3,534 | 3,140 | 3,113 | 3,442 | Nucleobase, nucleoside, nucleotide and nucleic acid metbolic process | GO:0006139 |
| 3,719 | 4,422 | 3,867 | 3,440 | Biological_process | GO:0008150 |
| 3,503 | 3,065 | 3,431 | 3,428 | Transport | GO:0006810 |
| 3,536 | 3,017 | 3,128 | 3,270 | Transferase activity | GO:0016740 |
| 2,604 | 2,787 | 2,859 | 2,873 | DNA metabolic process | GO:0006259 |
| 2,707 | 3,101 | 2,766 | 2,749 | Intracellular | GO:0005622 |
| 2,613 | 2,267 | 3,019 | 2,595 | Transportor activity | GO:0005215 |
| 1,978 | 2,436 | 1,986 | 2,044 | Binding | GO:0005488 |
| 1,800 | 1,669 | 1,844 | 1,893 | Carbohydrate metabolic process | GO:0005975 |
| 1,708 | 1,648 | 1,850 | 1,873 | Protein metabolic process | GO:0019538 |
| 2,276 | 1,810 | 1,903 | 1,800 | Cellular amino acid and derivative metabolic process | GO:0006519 |
| 1,811 | 1,605 | 1,631 | 1,692 | Biosynthetic process | GO:0009058 |
| 1,584 | 1,336 | 1,581 | 1,392 | Hydrolase activity | GO:0016787 |
| 1,153 | 1,352 | 967 | 1,326 | Ribosome | GO:0005840 |
| 1,125 | 1,060 | 1,423 | 1,129 | Regulation of biological process | GO:0050789 |
| 1,040 | 1,048 | 1,184 | 986 | Ion transport | GO:0006811 |
| 655 | 754 | 661 | 800 | Transcription | GO:0006350 |
| 764 | 971 | 830 | 787 | Nucleotide binding | GO:0000166 |
| 672 | 531 | 687 | 614 | Peptidase activity | GO:0008233 |
| 500 | 606 | 592 | 612 | Chromosome | GO:0005694 |
| 540 | 659 | 687 | 579 | DNA binding | GO:0003677 |
| 464 | 477 | 471 | 450 | Lipid metabolic process | GO:0006629 |
| 340 | 379 | 400 | 433 | Signal transduction | GO:0007165 |
| 346 | 284 | 353 | 432 | Response to stress | GO:0006950 |
| 352 | 412 | 328 | 427 | Structural molecule activity | GO:0005198 |
| 410 | 297 | 554 | 414 | Transcription factor activity | GO:0003700 |
| 425 | 524 | 466 | 407 | Protein binding | GO:0005515 |
| 306 | 290 | 332 | 390 | Protein complex | GO:0004234 |
| 300 | 191 | 337 | 376 | Kinase activity | GO:0016301 |
| 541 | 461 | 360 | 363 | Primary metabolic process | GO:0044238 |
| 307 | 336 | 402 | 361 | Protein transport | GO:0015031 |
| 524 | 554 | 340 | 306 | Generation of precursor metabolites and energy | GO:0006091 |
| 487 | 658 | 315 | 274 | Plasma membrane | GO:0005886 |
| 144 | 225 | 203 | 240 | Translation factor activity, nucleic acid binding | GO:0008135 |
| 182 | 181 | 225 | 233 | Protein modification process | GO:0006464 |
| 276 | 201 | 257 | 226 | External encapsulating structure | GO:0030312 |
| 91 | 245 | 160 | 185 | Regulation of gene expression, epigenetic | GO:0040029 |
| 128 | 168 | 162 | 178 | Transcription regulator activity | GO:0030528 |
| 175 | 322 | 158 | 176 | Cellular_component | GO:0005575 |
| 36 | 59 | 230 | 153 | Signal transducer activity | GO:0004871 |
| 151 | 237 | 177 | 152 | RNA binding | GO:0003723 |
| 92 | 134 | 160 | 147 | Cytosol | GO:0005829 |
| 108 | 126 | 135 | 144 | Cell wall | GO:0005618 |
| 84 | 110 | 88 | 132 | Nucleic acid binding | GO:0003676 |
| 276 | 228 | 149 | 124 | Cellular homeostasis | GO:0019725 |
| 166 | 171 | 151 | 104 | Nuclease activity | GO:0004518 |
| 142 | 161 | 129 | 93 | Cellular component organization | GO:0016043 |
| 34 | 64 | 66 | 87 | Anatomical structure morphogenesis | GO:0009653 |
| 51 | 65 | 66 | 82 | Organelle organization | GO:0006996 |
| 87 | 80 | 53 | 70 | Nuclear chromosome | GO:0000228 |
| 98 | 60 | 38 | 66 | Symbiosis, emcompassing mutualism through parasitism | GO:0044403 |
| 101 | 83 | 55 | 57 | Cell cycle | GO:0007049 |
| 104 | 93 | 73 | 43 | Catabolic process | GO:0009056 |
| 57 | 353 | 100 | 39 | Nucleus | GO:0005634 |
| 84 | 69 | 46 | 34 | Neurotransmitter transporter activity | GO:0005326 |
| 54 | 86 | 48 | 30 | Extracellular region | GO:0005576 |
| 34 | 21 | 42 | 30 | Electron carrier activity | GO:0009055 |
| 27 | 35 | 28 | 30 | Carbohydrate binding | GO:0030246 |
| 44 | 46 | 18 | 28 | Phosphoprotein phosphatase activity | GO:0004721 |
| 35 | 78 | 44 | 27 | Enzyme regulator activity | GO:0030234 |
| 64 | 218 | 59 | 23 | Receptor activity | GO:0004872 |
| 34 | 64 | 31 | 22 | Calcium ion binding | GO:0005509 |
| 3 | 27 | 91 | 18 | Plastid | GO:0009536 |
| 41 | 56 | 23 | 14 | Ion channel activity | GO:0005216 |
| 20 | 100 | 20 | 13 | Cytoskeleton | GO:0005856 |
| 10 | 17 | 24 | 12 | Lipid binding | GO:0008289 |
| 17 | 38 | 14 | 10 | Cytoplasmic membrane-bounded device | GO:0016023 |
| 13 | 14 | 13 | 7 | Cell death | GO:0008219 |
| 10 | 88 | 54 | 7 | Mitochondrion | GO:0005739 |
| 10 | 8 | 18 | 7 | Endoplasmic reticulum | GO:0005783 |
| 10 | 0 | 26 | 5 | Protein kinase activity | GO:0004672 |
| 10 | 3 | 8 | 5 | Proteinaceous extracellular matrix | GO:0005578 |
| 7 | 13 | 7 | 4 | Actin binding | GO:0003779 |
| 10 | 33 | 10 | 3 | Nucleoplasm | GO:0005654 |
| 3 | 29 | 4 | 3 | Extracellular space | GO:0005615 |

**Table S5. Physical locations and distribution of saliva-derived human SNPs on each of the four host genomes.**

| **SNP location** | **H105** | **H114** | **C201** | **C218** |
| --- | --- | --- | --- | --- |
| Intergenic | 59,579 | 368,456 | 300,135 | 177,968 |
| Intronic | 41,313 | 232,997 | 185,815 | 110,170 |
| Exonic | 1,485 | 7,341 | 5,673 | 3,320 |
| Upstream | 729 | 3,485 | 2,640 | 1,640 |
| Downstream | 667 | 3,578 | 2,732 | 1,557 |
| 5’-UTR | 199 | 904 | 636 | 392 |
| 3’-UTR | 994 | 5,123 | 3,927 | 2,307 |
| NcRNA | 2,355 | 13,478 | 10,997 | 6,611 |
| Splicing | 49 | 314 | 129 | 129 |
| Total SNPs | 107,370 | 635,676 | 512,684 | 304,094 |

**Table S6. Human SNPs with >30X sequence coverage (“SNPs with high read depth”) in each of the four saliva whole-ecosystem-sequencing dataset.**

|  | **Location** | **Gene** | **Chromosome** | **position** | **Reference** | **Alteration** | **Type** | **Quality** | **Coverage** | **Filter** |
| --- | --- | --- | --- | --- | --- | --- | --- | --- | --- | --- |
| **1** | **ncRNA_exonic** | **FLJ39739,LOC100286793** | **chr1** | **1.43E+08** | **G** | **C** | **heterozygosity** | **84** | **34** | **20** |
| **2** | **upstream** | **BMS1** | **chr10** | **42597006** | **G** | **T** | **homozygosity** | **99** | **79** | **19** |
| **3** | **upstream** | **BMS1** | **chr10** | **42597177** | **A** | **G** | **homozygosity** | **12.3** | **266** | **20** |
| **4** | **upstream** | **BMS1** | **chr10** | **42597784** | **A** | **G** | **homozygosity** | **122** | **79** | **16** |
| **5** | **upstream** | **BMS1** | **chr10** | **42597849** | **G** | **A** | **homozygosity** | **157** | **60** | **20** |
| **6** | **UTR5** | **BMS1** | **chr10** | **42598150** | **A** | **G** | **homozygosity** | **94.1** | **51** | **18** |
| **7** | **UTR5** | **BMS1** | **chr10** | **42598173** | **T** | **A** | **homozygosity** | **69** | **38** | **19** |
| **8** | **intronic** | **BMS1** | **chr10** | **42599160** | **A** | **G** | **heterozygosity** | **66** | **63** | **16** |
| **9** | **intronic** | **BMS1** | **chr10** | **42599603** | **A** | **G** | **homozygosity** | **144** | **273** | **20** |
| **10** | **intronic** | **BMS1** | **chr10** | **42599664** | **C** | **T** | **homozygosity** | **181** | **258** | **20** |
| **11** | **intronic** | **BMS1** | **chr10** | **42599680** | **C** | **A** | **heterozygosity** | **55** | **120** | **20** |
| **12** | **intronic** | **BMS1** | **chr10** | **42599725** | **A** | **T** | **homozygosity** | **130** | **46** | **18** |
| **13** | **intronic** | **BMS1** | **chr10** | **42599767** | **A** | **C** | **heterozygosity** | **36** | **304** | **20** |
| **14** | **UTR5** | **BMS1** | **chr10** | **42599836** | **T** | **C** | **homozygosity** | **149** | **90** | **20** |
| **15** | **exonic** | **BMS1** | **chr10** | **42599977** | **T** | **C** | **homozygosity** | **136** | **58** | **18** |
| **16** | **exonic** | **BMS1** | **chr10** | **42600006** | **A** | **G** | **heterozygosity** | **52** | **59** | **19** |
| **17** | **intronic** | **BMS1** | **chr10** | **42600054** | **T** | **C** | **homozygosity** | **101** | **31** | **18** |
| **18** | **intronic** | **BMS1** | **chr10** | **42600181** | **A** | **T** | **heterozygosity** | **105** | **44** | **20** |
| **19** | **ncRNA_intronic** | **LOC100129827** | **chr11** | **10530752** | **G** | **A** | **homozygosity** | **71.1** | **40** | **20** |
| **20** | **ncRNA_intronic** | **LOC100129827** | **chr11** | **10530850** | **A** | **G** | **homozygosity** | **161** | **33** | **20** |
| **21** | **intronic** | **SYTL2** | **chr11** | **85195075** | **T** | **C** | **homozygosity** | **118** | **64** | **20** |
| **22** | **intronic** | **SYTL2** | **chr11** | **85195154** | **A** | **T** | **homozygosity** | **201** | **73** | **20** |
| **23** | **intronic** | **MAPT** | **chr17** | **41400462** | **T** | **C** | **homozygosity** | **167** | **51** | **20** |
| **24** | **intronic** | **MAPT** | **chr17** | **41400511** | **C** | **G** | **homozygosity** | **160** | **36** | **20** |
| **25** | **intronic** | **MYST2** | **chr17** | **45234331** | **T** | **G** | **heterozygosity** | **4.13** | **36** | **20** |
| **26** | **intronic** | **MYST2** | **chr17** | **45234343** | **T** | **G** | **homozygosity** | **148** | **34** | **20** |
| **27** | **intronic** | **MYST2** | **chr17** | **45234360** | **A** | **C** | **homozygosity** | **137** | **32** | **20** |
| **28** | **intronic** | **MYST2** | **chr17** | **45249306** | **T** | **C** | **homozygosity** | **127** | **34** | **20** |
| **29** | **intronic** | **MYST2** | **chr17** | **45249316** | **T** | **C** | **homozygosity** | **148** | **38** | **20** |
| **30** | **intronic** | **MYST2** | **chr17** | **45249330** | **T** | **G** | **homozygosity** | **152** | **39** | **20** |
| **31** | **intronic** | **MYST2** | **chr17** | **45249365** | **C** | **T** | **homozygosity** | **167** | **32** | **20** |
| **32** | **ncRNA_intronic** | **ROCK1P1** | **chr18** | **108101** | **T** | **C** | **homozygosity** | **30.8** | **41** | **20** |
| **33** | **ncRNA_intronic** | **ROCK1P1** | **chr18** | **108116** | **T** | **G** | **heterozygosity** | **4.77** | **57** | **20** |
| **34** | **ncRNA_intronic** | **ROCK1P1** | **chr18** | **108128** | **C** | **A** | **heterozygosity** | **24** | **108** | **20** |
| **35** | **ncRNA_intronic** | **ROCK1P1** | **chr18** | **108131** | **T** | **C** | **heterozygosity** | **15.1** | **113** | **20** |
| **36** | **ncRNA_intronic** | **ROCK1P1** | **chr18** | **108165** | **G** | **T** | **heterozygosity** | **76** | **240** | **20** |
| **37** | **ncRNA_exonic** | **ROCK1P1** | **chr18** | **108309** | **T** | **A** | **heterozygosity** | **103** | **169** | **20** |
| **38** | **ncRNA_exonic** | **ROCK1P1** | **chr18** | **108329** | **A** | **G** | **homozygosity** | **108** | **57** | **20** |
| **39** | **ncRNA_exonic** | **ROCK1P1** | **chr18** | **108344** | **G** | **C** | **homozygosity** | **131** | **39** | **20** |
| **40** | **ncRNA_exonic** | **ROCK1P1** | **chr18** | **108358** | **C** | **A** | **heterozygosity** | **73** | **45** | **20** |
| **41** | **ncRNA_splicing** | **ROCK1P1** | **chr18** | **108505** | **G** | **A** | **heterozygosity** | **99** | **33** | **20** |
| **42** | **ncRNA_intronic** | **ROCK1P1** | **chr18** | **110338** | **C** | **A** | **heterozygosity** | **37** | **38** | **20** |
| **43** | **ncRNA_intronic** | **ROCK1P1** | **chr18** | **110425** | **G** | **T** | **heterozygosity** | **89** | **31** | **20** |
| **44** | **ncRNA_intronic** | **ROCK1P1** | **chr18** | **110627** | **C** | **T** | **heterozygosity** | **52** | **38** | **20** |
| **45** | **intronic** | **TNRC6B** | **chr22** | **39040460** | **T** | **C** | **heterozygosity** | **68.1** | **34** | **14** |
| **46** | **intronic** | **ACAP2** | **chr3** | **1.97E+08** | **G** | **A** | **heterozygosity** | **21** | **58** | **17** |
| **47** | **intronic** | **ACAP2** | **chr3** | **1.97E+08** | **C** | **T** | **heterozygosity** | **78** | **72** | **18** |
| **48** | **intronic** | **ACAP2** | **chr3** | **1.97E+08** | **A** | **T** | **heterozygosity** | **43** | **60** | **19** |
| **49** | **intronic** | **TXNDC15** | **chr5** | **1.34E+08** | **C** | **T** | **homozygosity** | **117** | **35** | **20** |
| **50** | **intronic** | **TXNDC15** | **chr5** | **1.34E+08** | **A** | **G** | **homozygosity** | **97** | **36** | **20** |
| **51** | **intronic** | **TXNDC15** | **chr5** | **1.34E+08** | **A** | **G** | **homozygosity** | **112** | **52** | **20** |
| **52** | **intronic** | **TXNDC15** | **chr5** | **1.34E+08** | **T** | **C** | **homozygosity** | **140** | **67** | **20** |
| **53** | **intronic** | **TXNDC15** | **chr5** | **1.34E+08** | **C** | **T** | **homozygosity** | **169** | **75** | **20** |
| **54** | **intronic** | **TXNDC15** | **chr5** | **1.34E+08** | **A** | **G** | **homozygosity** | **183** | **80** | **20** |
| **55** | **intronic** | **TXNDC15** | **chr5** | **1.34E+08** | **A** | **G** | **homozygosity** | **194** | **73** | **20** |
| **56** | **intronic** | **TXNDC15** | **chr5** | **1.34E+08** | **C** | **T** | **homozygosity** | **127** | **40** | **20** |
| **57** | **intronic** | **TXNDC15** | **chr5** | **1.34E+08** | **G** | **A** | **homozygosity** | **136** | **35** | **20** |
| **58** | **intronic** | **TXNDC15** | **chr5** | **1.34E+08** | **G** | **A** | **homozygosity** | **119** | **62** | **20** |
| **59** | **intronic** | **TXNDC15** | **chr5** | **1.34E+08** | **C** | **T** | **homozygosity** | **146** | **87** | **20** |
| **60** | **intronic** | **TXNDC15** | **chr5** | **1.34E+08** | **G** | **A** | **homozygosity** | **147** | **106** | **20** |
| **61** | **intronic** | **TXNDC15** | **chr5** | **1.34E+08** | **C** | **T** | **homozygosity** | **170** | **103** | **20** |
| **62** | **intronic** | **TXNDC15** | **chr5** | **1.34E+08** | **T** | **C** | **homozygosity** | **167** | **98** | **20** |
| **63** | **intronic** | **TXNDC15** | **chr5** | **1.34E+08** | **A** | **G** | **homozygosity** | **181** | **122** | **20** |
| **64** | **intronic** | **TXNDC15** | **chr5** | **1.34E+08** | **G** | **A** | **homozygosity** | **183** | **117** | **20** |
| **65** | **intronic** | **TXNDC15** | **chr5** | **1.34E+08** | **T** | **C** | **homozygosity** | **178** | **123** | **20** |
| **66** | **intronic** | **TXNDC15** | **chr5** | **1.34E+08** | **T** | **C** | **homozygosity** | **174** | **131** | **20** |
| **67** | **intronic** | **TXNDC15** | **chr5** | **1.34E+08** | **G** | **A** | **homozygosity** | **182** | **145** | **20** |
| **68** | **intronic** | **TXNDC15** | **chr5** | **1.34E+08** | **A** | **G** | **homozygosity** | **179** | **138** | **20** |
| **69** | **intronic** | **TXNDC15** | **chr5** | **1.34E+08** | **G** | **A** | **homozygosity** | **167** | **136** | **20** |
| **70** | **intronic** | **TXNDC15** | **chr5** | **1.34E+08** | **C** | **T** | **homozygosity** | **174** | **138** | **20** |
| **71** | **intronic** | **TXNDC15** | **chr5** | **1.34E+08** | **G** | **A** | **homozygosity** | **178** | **99** | **20** |
| **72** | **intronic** | **TXNDC15** | **chr5** | **1.34E+08** | **G** | **T** | **homozygosity** | **166** | **88** | **20** |
| **73** | **intronic** | **TXNDC15** | **chr5** | **1.34E+08** | **T** | **C** | **homozygosity** | **156** | **72** | **20** |
| **74** | **intronic** | **TXNDC15** | **chr5** | **1.34E+08** | **C** | **T** | **homozygosity** | **158** | **66** | **20** |
| **75** | **intronic** | **TXNDC15** | **chr5** | **1.34E+08** | **G** | **A** | **homozygosity** | **162** | **61** | **20** |
| **76** | **intronic** | **TXNDC15** | **chr5** | **1.34E+08** | **C** | **T** | **homozygosity** | **163** | **63** | **20** |
| **77** | **intronic** | **TXNDC15** | **chr5** | **1.34E+08** | **A** | **T** | **homozygosity** | **162** | **42** | **20** |
| **78** | **intronic** | **TXNDC15** | **chr5** | **1.34E+08** | **A** | **G** | **homozygosity** | **136** | **44** | **20** |
| **79** | **intronic** | **TXNDC15** | **chr5** | **1.34E+08** | **C** | **A** | **homozygosity** | **150** | **48** | **20** |
| **80** | **intronic** | **TXNDC15** | **chr5** | **1.34E+08** | **G** | **A** | **homozygosity** | **143** | **61** | **20** |
| **81** | **intronic** | **TXNDC15** | **chr5** | **1.34E+08** | **G** | **A** | **homozygosity** | **172** | **67** | **20** |
| **82** | **intronic** | **TXNDC15** | **chr5** | **1.34E+08** | **A** | **G** | **homozygosity** | **163** | **63** | **20** |
| **83** | **intronic** | **TXNDC15** | **chr5** | **1.34E+08** | **G** | **A** | **homozygosity** | **166** | **53** | **20** |
| **84** | **intronic** | **TXNDC15** | **chr5** | **1.34E+08** | **A** | **G** | **homozygosity** | **164** | **54** | **20** |
| **85** | **intronic** | **TXNDC15** | **chr5** | **1.34E+08** | **A** | **G** | **homozygosity** | **157** | **60** | **20** |
| **86** | **intronic** | **TXNDC15** | **chr5** | **1.34E+08** | **C** | **G** | **homozygosity** | **134** | **56** | **20** |
| **87** | **intronic** | **TXNDC15** | **chr5** | **1.34E+08** | **C** | **T** | **homozygosity** | **141** | **66** | **20** |
| **88** | **intronic** | **TXNDC15** | **chr5** | **1.34E+08** | **T** | **C** | **homozygosity** | **153** | **77** | **20** |
| **89** | **intronic** | **TXNDC15** | **chr5** | **1.34E+08** | **T** | **C** | **homozygosity** | **153** | **119** | **20** |
| **90** | **intronic** | **TXNDC15** | **chr5** | **1.34E+08** | **T** | **C** | **homozygosity** | **169** | **127** | **20** |
| **91** | **intronic** | **TXNDC15** | **chr5** | **1.34E+08** | **T** | **C** | **homozygosity** | **181** | **133** | **20** |
| **92** | **intronic** | **TXNDC15** | **chr5** | **1.34E+08** | **A** | **G** | **homozygosity** | **185** | **107** | **20** |
| **93** | **intronic** | **TXNDC15** | **chr5** | **1.34E+08** | **T** | **C** | **homozygosity** | **187** | **107** | **20** |
| **94** | **intronic** | **TXNDC15** | **chr5** | **1.34E+08** | **G** | **A** | **homozygosity** | **131** | **101** | **20** |
| **95** | **intronic** | **TXNDC15** | **chr5** | **1.34E+08** | **G** | **A** | **homozygosity** | **166** | **98** | **20** |
| **96** | **intronic** | **TXNDC15** | **chr5** | **1.34E+08** | **A** | **G** | **homozygosity** | **156** | **94** | **20** |
| **97** | **intronic** | **TXNDC15** | **chr5** | **1.34E+08** | **G** | **A** | **homozygosity** | **154** | **100** | **20** |
| **98** | **intronic** | **TXNDC15** | **chr5** | **1.34E+08** | **A** | **G** | **homozygosity** | **157** | **107** | **20** |
| **99** | **intronic** | **TXNDC15** | **chr5** | **1.34E+08** | **C** | **T** | **homozygosity** | **186** | **162** | **20** |
| **100** | **intronic** | **TXNDC15** | **chr5** | **1.34E+08** | **G** | **A** | **homozygosity** | **205** | **201** | **20** |
| **101** | **intronic** | **TXNDC15** | **chr5** | **1.34E+08** | **C** | **T** | **homozygosity** | **219** | **220** | **20** |
| **102** | **intronic** | **TXNDC15** | **chr5** | **1.34E+08** | **C** | **T** | **homozygosity** | **218** | **198** | **20** |
| **103** | **intronic** | **TXNDC15** | **chr5** | **1.34E+08** | **A** | **G** | **homozygosity** | **222** | **178** | **20** |
| **104** | **intronic** | **TXNDC15** | **chr5** | **1.34E+08** | **A** | **G** | **homozygosity** | **197** | **111** | **20** |
| **105** | **intronic** | **TXNDC15** | **chr5** | **1.34E+08** | **A** | **G** | **homozygosity** | **195** | **95** | **20** |
| **106** | **exonic** | **TXNDC15** | **chr5** | **1.34E+08** | **G** | **A** | **homozygosity** | **80** | **36** | **20** |
| **107** | **exonic** | **TXNDC15** | **chr5** | **1.34E+08** | **G** | **A** | **homozygosity** | **88** | **40** | **20** |
| **108** | **exonic** | **TXNDC15** | **chr5** | **1.34E+08** | **G** | **A** | **homozygosity** | **115** | **60** | **20** |
| **109** | **UTR3** | **TXNDC15** | **chr5** | **1.34E+08** | **A** | **G** | **homozygosity** | **141** | **96** | **20** |
| **110** | **UTR3** | **TXNDC15** | **chr5** | **1.34E+08** | **T** | **C** | **homozygosity** | **157** | **103** | **20** |
| **111** | **UTR3** | **TXNDC15** | **chr5** | **1.34E+08** | **G** | **A** | **homozygosity** | **151** | **116** | **20** |
| **112** | **UTR3** | **TXNDC15** | **chr5** | **1.34E+08** | **A** | **G** | **homozygosity** | **191** | **142** | **20** |
| **113** | **UTR3** | **TXNDC15** | **chr5** | **1.34E+08** | **C** | **T** | **homozygosity** | **198** | **148** | **20** |
| **114** | **UTR3** | **TXNDC15** | **chr5** | **1.34E+08** | **T** | **C** | **homozygosity** | **209** | **86** | **20** |
| **115** | **UTR3** | **TXNDC15** | **chr5** | **1.34E+08** | **C** | **T** | **homozygosity** | **84** | **41** | **20** |
| **116** | **UTR3** | **TXNDC15** | **chr5** | **1.34E+08** | **G** | **A** | **homozygosity** | **79** | **35** | **20** |
| **117** | **UTR3** | **TXNDC15** | **chr5** | **1.34E+08** | **G** | **A** | **homozygosity** | **75** | **48** | **20** |
| **118** | **UTR3** | **TXNDC15** | **chr5** | **1.34E+08** | **A** | **G** | **homozygosity** | **117** | **77** | **20** |
| **119** | **UTR3** | **TXNDC15** | **chr5** | **1.34E+08** | **G** | **A** | **homozygosity** | **177** | **126** | **20** |
| **120** | **UTR3** | **TXNDC15** | **chr5** | **1.34E+08** | **A** | **G** | **homozygosity** | **203** | **131** | **20** |
| **121** | **UTR3** | **TXNDC15** | **chr5** | **1.34E+08** | **C** | **T** | **homozygosity** | **201** | **95** | **20** |
| **122** | **UTR3** | **TXNDC15** | **chr5** | **1.34E+08** | **C** | **T** | **homozygosity** | **114** | **52** | **20** |
| **123** | **UTR3** | **TXNDC15** | **chr5** | **1.34E+08** | **T** | **G** | **homozygosity** | **154** | **44** | **20** |
| **124** | **UTR3** | **TXNDC15** | **chr5** | **1.34E+08** | **A** | **G** | **homozygosity** | **163** | **44** | **20** |
| **125** | **intronic** | **SULF1** | **chr8** | **70602341** | **A** | **G** | **homozygosity** | **142** | **86** | **20** |
| **126** | **ncRNA_intronic** | **PGM5P2** | **chr9** | **68424163** | **T** | **G** | **homozygosity** | **134** | **31** | **20** |
| **127** | **ncRNA_intronic** | **PGM5P2** | **chr9** | **68424180** | **C** | **G** | **heterozygosity** | **42** | **31** | **20** |
| **128** | **ncRNA_intronic** | **PGM5P2** | **chr9** | **68424213** | **C** | **T** | **heterozygosity** | **16.1** | **37** | **20** |
| **129** | **ncRNA_intronic** | **PGM5P2** | **chr9** | **68424243** | **C** | **A** | **heterozygosity** | **85** | **32** | **20** |
| **130** | **ncRNA_intronic** | **PGM5P2** | **chr9** | **68426181** | **C** | **T** | **heterozygosity** | **6.2** | **34** | **20** |
| **131** | **ncRNA_intronic** | **PGM5P2** | **chr9** | **68426195** | **C** | **G** | **heterozygosity** | **54** | **39** | **20** |
| **132** | **ncRNA_intronic** | **PGM5P2** | **chr9** | **68426229** | **A** | **G** | **heterozygosity** | **3.01** | **38** | **20** |
| **133** | **ncRNA_intronic** | **PGM5P2** | **chr9** | **68426352** | **C** | **T** | **heterozygosity** | **7.8** | **35** | **20** |
| **134** | **intronic** | **USP9Y** | **chrY** | **13464951** | **A** | **G** | **heterozygosity** | **44.3** | **39** | **19** |
| **135** | **intronic** | **USP9Y** | **chrY** | **13464966** | **T** | **C** | **homozygosity** | **91** | **43** | **19** |
| **1** | **upstream** | **BMS1** | **chr10** | **42596997** | **A** | **G** | **heterozygosity** | **94** | **38** | **20** |
| **2** | **upstream** | **BMS1** | **chr10** | **42597006** | **G** | **T** | **homozygosity** | **139** | **39** | **20** |
| **3** | **upstream** | **BMS1** | **chr10** | **42597177** | **A** | **G** | **heterozygosity** | **28** | **140** | **20** |
| **4** | **intronic** | **BMS1** | **chr10** | **42599566** | **C** | **G** | **homozygosity** | **77** | **68** | **20** |
| **5** | **intronic** | **BMS1** | **chr10** | **42599603** | **A** | **G** | **homozygosity** | **178** | **207** | **20** |
| **6** | **intronic** | **BMS1** | **chr10** | **42599664** | **C** | **T** | **homozygosity** | **175** | **157** | **20** |
| **7** | **intronic** | **BMS1** | **chr10** | **42599680** | **C** | **A** | **heterozygosity** | **42** | **79** | **20** |
| **8** | **UTR5** | **BMS1** | **chr10** | **42599836** | **T** | **C** | **heterozygosity** | **95** | **78** | **20** |
| **9** | **intronic** | **BMS1** | **chr10** | **42600181** | **A** | **T** | **homozygosity** | **125** | **54** | **20** |
| **10** | **ncRNA_intronic** | **LOC100129827** | **chr11** | **10530850** | **A** | **G** | **homozygosity** | **131** | **40** | **20** |
| **11** | **intronic** | **SYTL2** | **chr11** | **85195075** | **T** | **C** | **homozygosity** | **147** | **59** | **20** |
| **12** | **intronic** | **SYTL2** | **chr11** | **85195154** | **A** | **T** | **homozygosity** | **173** | **40** | **20** |
| **13** | **intronic** | **CARKD** | **chr13** | **1.10E+08** | **T** | **C** | **homozygosity** | **160** | **40** | **20** |
| **14** | **intronic** | **CARKD** | **chr13** | **1.10E+08** | **A** | **G** | **homozygosity** | **114** | **31** | **20** |
| **15** | **intronic** | **MYST2** | **chr17** | **45234343** | **T** | **G** | **heterozygosity** | **112** | **39** | **20** |
| **16** | **intronic** | **MYST2** | **chr17** | **45234360** | **A** | **C** | **heterozygosity** | **112** | **40** | **20** |
| **17** | **intronic** | **MYST2** | **chr17** | **45249306** | **T** | **C** | **homozygosity** | **118** | **36** | **20** |
| **18** | **intronic** | **MYST2** | **chr17** | **45249316** | **T** | **C** | **homozygosity** | **126** | **36** | **20** |
| **19** | **intronic** | **MYST2** | **chr17** | **45249330** | **T** | **G** | **homozygosity** | **146** | **34** | **20** |
| **20** | **ncRNA_intronic** | **ROCK1P1** | **chr18** | **108128** | **C** | **A** | **heterozygosity** | **11.3** | **32** | **20** |
| **21** | **ncRNA_intronic** | **ROCK1P1** | **chr18** | **108131** | **T** | **C** | **heterozygosity** | **8.64** | **34** | **20** |
| **22** | **ncRNA_intronic** | **ROCK1P1** | **chr18** | **108165** | **G** | **T** | **heterozygosity** | **81** | **103** | **20** |
| **23** | **ncRNA_exonic** | **ROCK1P1** | **chr18** | **108309** | **T** | **A** | **homozygosity** | **129** | **57** | **20** |
| **24** | **ncRNA_exonic** | **ROCK1P1** | **chr18** | **108329** | **A** | **G** | **homozygosity** | **95.5** | **38** | **20** |
| **25** | **intronic** | **TNRC6B** | **chr22** | **39040427** | **A** | **G** | **homozygosity** | **91** | **32** | **13** |
| **26** | **intronic** | **ACAP2** | **chr3** | **1.97E+08** | **A** | **T** | **heterozygosity** | **28** | **35** | **20** |
| **27** | **intronic** | **TXNDC15** | **chr5** | **1.34E+08** | **A** | **G** | **homozygosity** | **109** | **37** | **20** |
| **28** | **intronic** | **TXNDC15** | **chr5** | **1.34E+08** | **C** | **T** | **homozygosity** | **111** | **36** | **20** |
| **29** | **intronic** | **TXNDC15** | **chr5** | **1.34E+08** | **C** | **T** | **homozygosity** | **124** | **64** | **20** |
| **30** | **intronic** | **TXNDC15** | **chr5** | **1.34E+08** | **A** | **G** | **homozygosity** | **96** | **66** | **20** |
| **31** | **intronic** | **TXNDC15** | **chr5** | **1.34E+08** | **A** | **G** | **homozygosity** | **162** | **81** | **20** |
| **32** | **intronic** | **TXNDC15** | **chr5** | **1.34E+08** | **T** | **C** | **homozygosity** | **180** | **94** | **20** |
| **33** | **intronic** | **TXNDC15** | **chr5** | **1.34E+08** | **C** | **T** | **homozygosity** | **190** | **90** | **20** |
| **34** | **intronic** | **TXNDC15** | **chr5** | **1.34E+08** | **A** | **G** | **homozygosity** | **193** | **90** | **20** |
| **35** | **intronic** | **TXNDC15** | **chr5** | **1.34E+08** | **A** | **G** | **homozygosity** | **192** | **97** | **20** |
| **36** | **exonic** | **TXNDC15** | **chr5** | **1.34E+08** | **T** | **C** | **homozygosity** | **157** | **36** | **20** |
| **37** | **intronic** | **TXNDC15** | **chr5** | **1.34E+08** | **G** | **A** | **homozygosity** | **166** | **33** | **20** |
| **38** | **intronic** | **TXNDC15** | **chr5** | **1.34E+08** | **A** | **G** | **homozygosity** | **93** | **36** | **20** |
| **39** | **intronic** | **TXNDC15** | **chr5** | **1.34E+08** | **C** | **T** | **homozygosity** | **126** | **52** | **20** |
| **40** | **intronic** | **TXNDC15** | **chr5** | **1.34E+08** | **G** | **A** | **homozygosity** | **161** | **55** | **20** |
| **41** | **intronic** | **TXNDC15** | **chr5** | **1.34E+08** | **C** | **A** | **homozygosity** | **69** | **43** | **20** |
| **42** | **intronic** | **TXNDC15** | **chr5** | **1.34E+08** | **A** | **T** | **homozygosity** | **69** | **42** | **20** |
| **43** | **intronic** | **TXNDC15** | **chr5** | **1.34E+08** | **A** | **G** | **homozygosity** | **147** | **48** | **20** |
| **44** | **intronic** | **TXNDC15** | **chr5** | **1.34E+08** | **A** | **G** | **homozygosity** | **157** | **36** | **20** |
| **45** | **intronic** | **TXNDC15** | **chr5** | **1.34E+08** | **G** | **A** | **homozygosity** | **159** | **35** | **20** |
| **46** | **intronic** | **TXNDC15** | **chr5** | **1.34E+08** | **G** | **A** | **homozygosity** | **147** | **73** | **20** |
| **47** | **intronic** | **TXNDC15** | **chr5** | **1.34E+08** | **C** | **T** | **homozygosity** | **159** | **96** | **20** |
| **48** | **intronic** | **TXNDC15** | **chr5** | **1.34E+08** | **G** | **A** | **homozygosity** | **160** | **115** | **20** |
| **49** | **intronic** | **TXNDC15** | **chr5** | **1.34E+08** | **C** | **T** | **homozygosity** | **184** | **103** | **20** |
| **50** | **intronic** | **TXNDC15** | **chr5** | **1.34E+08** | **T** | **C** | **homozygosity** | **183** | **107** | **20** |
| **51** | **intronic** | **TXNDC15** | **chr5** | **1.34E+08** | **A** | **G** | **homozygosity** | **175** | **99** | **20** |
| **52** | **intronic** | **TXNDC15** | **chr5** | **1.34E+08** | **G** | **A** | **homozygosity** | **177** | **94** | **20** |
| **53** | **intronic** | **TXNDC15** | **chr5** | **1.34E+08** | **T** | **C** | **homozygosity** | **179** | **81** | **20** |
| **54** | **intronic** | **TXNDC15** | **chr5** | **1.34E+08** | **T** | **C** | **homozygosity** | **182** | **78** | **20** |
| **55** | **intronic** | **TXNDC15** | **chr5** | **1.34E+08** | **C** | **T** | **homozygosity** | **140** | **33** | **20** |
| **56** | **intronic** | **TXNDC15** | **chr5** | **1.34E+08** | **G** | **A** | **homozygosity** | **174** | **72** | **20** |
| **57** | **intronic** | **TXNDC15** | **chr5** | **1.34E+08** | **G** | **T** | **homozygosity** | **169** | **67** | **20** |
| **58** | **intronic** | **TXNDC15** | **chr5** | **1.34E+08** | **T** | **C** | **homozygosity** | **177** | **57** | **20** |
| **59** | **intronic** | **TXNDC15** | **chr5** | **1.34E+08** | **C** | **T** | **homozygosity** | **180** | **53** | **20** |
| **60** | **intronic** | **TXNDC15** | **chr5** | **1.34E+08** | **G** | **A** | **homozygosity** | **179** | **46** | **20** |
| **61** | **intronic** | **TXNDC15** | **chr5** | **1.34E+08** | **C** | **T** | **homozygosity** | **177** | **40** | **20** |
| **62** | **intronic** | **TXNDC15** | **chr5** | **1.34E+08** | **C** | **A** | **homozygosity** | **154** | **31** | **20** |
| **63** | **intronic** | **TXNDC15** | **chr5** | **1.34E+08** | **G** | **A** | **homozygosity** | **153** | **68** | **20** |
| **64** | **intronic** | **TXNDC15** | **chr5** | **1.34E+08** | **G** | **A** | **homozygosity** | **175** | **99** | **20** |
| **65** | **intronic** | **TXNDC15** | **chr5** | **1.34E+08** | **A** | **G** | **homozygosity** | **182** | **102** | **20** |
| **66** | **intronic** | **TXNDC15** | **chr5** | **1.34E+08** | **G** | **A** | **homozygosity** | **188** | **71** | **20** |
| **67** | **intronic** | **TXNDC15** | **chr5** | **1.34E+08** | **A** | **G** | **homozygosity** | **191** | **71** | **20** |
| **68** | **intronic** | **TXNDC15** | **chr5** | **1.34E+08** | **A** | **G** | **homozygosity** | **187** | **69** | **20** |
| **69** | **intronic** | **TXNDC15** | **chr5** | **1.34E+08** | **C** | **G** | **homozygosity** | **171** | **41** | **20** |
| **70** | **intronic** | **TXNDC15** | **chr5** | **1.34E+08** | **C** | **T** | **homozygosity** | **174** | **43** | **20** |
| **71** | **intronic** | **TXNDC15** | **chr5** | **1.34E+08** | **T** | **C** | **homozygosity** | **169** | **49** | **20** |
| **72** | **intronic** | **TXNDC15** | **chr5** | **1.34E+08** | **T** | **C** | **homozygosity** | **161** | **106** | **20** |
| **73** | **intronic** | **TXNDC15** | **chr5** | **1.34E+08** | **G** | **A** | **homozygosity** | **167** | **125** | **20** |
| **74** | **intronic** | **TXNDC15** | **chr5** | **1.34E+08** | **T** | **C** | **homozygosity** | **198** | **171** | **20** |
| **75** | **intronic** | **TXNDC15** | **chr5** | **1.34E+08** | **T** | **C** | **homozygosity** | **201** | **161** | **20** |
| **76** | **intronic** | **TXNDC15** | **chr5** | **1.34E+08** | **A** | **G** | **homozygosity** | **204** | **114** | **20** |
| **77** | **intronic** | **TXNDC15** | **chr5** | **1.34E+08** | **T** | **C** | **homozygosity** | **204** | **104** | **20** |
| **78** | **intronic** | **TXNDC15** | **chr5** | **1.34E+08** | **G** | **A** | **homozygosity** | **145** | **75** | **20** |
| **79** | **intronic** | **TXNDC15** | **chr5** | **1.34E+08** | **G** | **A** | **homozygosity** | **174** | **64** | **20** |
| **80** | **intronic** | **TXNDC15** | **chr5** | **1.34E+08** | **T** | **C** | **homozygosity** | **160** | **59** | **20** |
| **81** | **intronic** | **TXNDC15** | **chr5** | **1.34E+08** | **A** | **G** | **homozygosity** | **157** | **54** | **20** |
| **82** | **intronic** | **TXNDC15** | **chr5** | **1.34E+08** | **G** | **A** | **homozygosity** | **140** | **72** | **20** |
| **83** | **intronic** | **TXNDC15** | **chr5** | **1.34E+08** | **A** | **G** | **homozygosity** | **143** | **77** | **20** |
| **84** | **intronic** | **TXNDC15** | **chr5** | **1.34E+08** | **C** | **T** | **homozygosity** | **188** | **157** | **20** |
| **85** | **intronic** | **TXNDC15** | **chr5** | **1.34E+08** | **G** | **A** | **homozygosity** | **203** | **203** | **20** |
| **86** | **intronic** | **TXNDC15** | **chr5** | **1.34E+08** | **C** | **T** | **homozygosity** | **222** | **256** | **20** |
| **87** | **intronic** | **TXNDC15** | **chr5** | **1.34E+08** | **C** | **T** | **homozygosity** | **222** | **238** | **20** |
| **88** | **intronic** | **TXNDC15** | **chr5** | **1.34E+08** | **A** | **G** | **homozygosity** | **222** | **200** | **20** |
| **89** | **intronic** | **TXNDC15** | **chr5** | **1.34E+08** | **A** | **G** | **homozygosity** | **206** | **111** | **20** |
| **90** | **intronic** | **TXNDC15** | **chr5** | **1.34E+08** | **A** | **G** | **homozygosity** | **195** | **90** | **20** |
| **91** | **exonic** | **TXNDC15** | **chr5** | **1.34E+08** | **G** | **A** | **homozygosity** | **88** | **41** | **20** |
| **92** | **exonic** | **TXNDC15** | **chr5** | **1.34E+08** | **G** | **A** | **homozygosity** | **94** | **48** | **20** |
| **93** | **exonic** | **TXNDC15** | **chr5** | **1.34E+08** | **G** | **A** | **homozygosity** | **136** | **86** | **20** |
| **94** | **UTR3** | **TXNDC15** | **chr5** | **1.34E+08** | **A** | **G** | **homozygosity** | **185** | **140** | **20** |
| **95** | **UTR3** | **TXNDC15** | **chr5** | **1.34E+08** | **T** | **C** | **homozygosity** | **196** | **154** | **20** |
| **96** | **UTR3** | **TXNDC15** | **chr5** | **1.34E+08** | **G** | **A** | **homozygosity** | **197** | **148** | **20** |
| **97** | **UTR3** | **TXNDC15** | **chr5** | **1.34E+08** | **A** | **G** | **homozygosity** | **194** | **123** | **20** |
| **98** | **UTR3** | **TXNDC15** | **chr5** | **1.34E+08** | **C** | **T** | **homozygosity** | **188** | **117** | **20** |
| **99** | **UTR3** | **TXNDC15** | **chr5** | **1.34E+08** | **G** | **A** | **homozygosity** | **180** | **107** | **20** |
| **100** | **UTR3** | **TXNDC15** | **chr5** | **1.34E+08** | **T** | **C** | **homozygosity** | **202** | **87** | **20** |
| **101** | **UTR3** | **TXNDC15** | **chr5** | **1.34E+08** | **C** | **T** | **homozygosity** | **130** | **39** | **20** |
| **102** | **UTR3** | **TXNDC15** | **chr5** | **1.34E+08** | **G** | **A** | **homozygosity** | **88** | **40** | **20** |
| **103** | **UTR3** | **TXNDC15** | **chr5** | **1.34E+08** | **A** | **G** | **homozygosity** | **143** | **82** | **20** |
| **104** | **UTR3** | **TXNDC15** | **chr5** | **1.34E+08** | **G** | **A** | **homozygosity** | **181** | **135** | **20** |
| **105** | **UTR3** | **TXNDC15** | **chr5** | **1.34E+08** | **A** | **G** | **homozygosity** | **210** | **153** | **20** |
| **106** | **UTR3** | **TXNDC15** | **chr5** | **1.34E+08** | **C** | **T** | **homozygosity** | **214** | **101** | **20** |
| **107** | **UTR3** | **TXNDC15** | **chr5** | **1.34E+08** | **C** | **T** | **homozygosity** | **137** | **51** | **20** |
| **108** | **UTR3** | **TXNDC15** | **chr5** | **1.34E+08** | **G** | **A** | **homozygosity** | **121** | **40** | **20** |
| **109** | **UTR3** | **TXNDC15** | **chr5** | **1.34E+08** | **T** | **C** | **homozygosity** | **125** | **55** | **20** |
| **110** | **UTR3** | **TXNDC15** | **chr5** | **1.34E+08** | **T** | **C** | **homozygosity** | **129** | **57** | **20** |
| **111** | **UTR3** | **TXNDC15** | **chr5** | **1.34E+08** | **C** | **G** | **homozygosity** | **154** | **101** | **20** |
| **112** | **UTR3** | **TXNDC15** | **chr5** | **1.34E+08** | **T** | **G** | **homozygosity** | **201** | **153** | **20** |
| **113** | **UTR3** | **TXNDC15** | **chr5** | **1.34E+08** | **A** | **G** | **homozygosity** | **199** | **146** | **20** |
| **114** | **UTR3** | **TXNDC15** | **chr5** | **1.34E+08** | **G** | **A** | **homozygosity** | **182** | **47** | **20** |
| **115** | **intronic** | **SULF1** | **chr8** | **70602341** | **A** | **G** | **homozygosity** | **122** | **45** | **20** |
| **1** | **s** | **BMS1** | **chr10** | **42596997** | **A** | **G** | **heterozygosity** | **54** | **70** | **20** |
| **2** | **upstream** | **BMS1** | **chr10** | **42597006** | **G** | **T** | **homozygosity** | **137** | **58** | **20** |
| **3** | **upstream** | **BMS1** | **chr10** | **42597106** | **A** | **G** | **homozygosity** | **87.1** | **324** | **20** |
| **4** | **upstream** | **BMS1** | **chr10** | **42597177** | **A** | **G** | **homozygosity** | **69** | **97** | **20** |
| **5** | **upstream** | **BMS1** | **chr10** | **42597186** | **C** | **T** | **heterozygosity** | **37** | **88** | **20** |
| **6** | **upstream** | **BMS1** | **chr10** | **42597849** | **G** | **A** | **homozygosity** | **126** | **32** | **19** |
| **7** | **intronic** | **BMS1** | **chr10** | **42599566** | **C** | **G** | **homozygosity** | **95** | **180** | **20** |
| **8** | **intronic** | **BMS1** | **chr10** | **42599603** | **A** | **G** | **homozygosity** | **156** | **748** | **20** |
| **9** | **intronic** | **BMS1** | **chr10** | **42599664** | **C** | **T** | **homozygosity** | **128** | **476** | **20** |
| **10** | **intronic** | **BMS1** | **chr10** | **42599680** | **C** | **A** | **heterozygosity** | **108** | **264** | **20** |
| **11** | **intronic** | **BMS1** | **chr10** | **42599725** | **A** | **T** | **homozygosity** | **172** | **151** | **20** |
| **12** | **UTR5** | **BMS1** | **chr10** | **42599836** | **T** | **C** | **homozygosity** | **159** | **118** | **20** |
| **13** | **exonic** | **BMS1** | **chr10** | **42599977** | **T** | **C** | **homozygosity** | **169** | **126** | **20** |
| **14** | **exonic** | **BMS1** | **chr10** | **42600006** | **A** | **G** | **heterozygosity** | **47** | **66** | **20** |
| **15** | **intronic** | **BMS1** | **chr10** | **42600054** | **T** | **C** | **heterozygosity** | **40** | **72** | **20** |
| **16** | **intronic** | **BMS1** | **chr10** | **42600080** | **C** | **T** | **heterozygosity** | **17.1** | **33** | **20** |
| **17** | **intronic** | **BMS1** | **chr10** | **42600181** | **A** | **T** | **heterozygosity** | **70** | **134** | **20** |
| **18** | **intronic** | **BMS1** | **chr10** | **42600224** | **T** | **C** | **heterozygosity** | **79** | **91** | **20** |
| **19** | **intronic** | **BMS1** | **chr10** | **42600229** | **A** | **G** | **heterozygosity** | **41** | **67** | **20** |
| **20** | **intronic** | **BMS1** | **chr10** | **42600352** | **G** | **A** | **heterozygosity** | **8.64** | **54** | **20** |
| **21** | **intronic** | **GABRG3** | **chr15** | **25008470** | **A** | **G** | **heterozygosity** | **108** | **51** | **20** |
| **22** | **ncRNA_intronic** | **ROCK1P1** | **chr18** | **108101** | **T** | **C** | **homozygosity** | **136** | **63** | **20** |
| **23** | **ncRNA_intronic** | **ROCK1P1** | **chr18** | **108116** | **T** | **G** | **heterozygosity** | **33** | **69** | **20** |
| **24** | **ncRNA_intronic** | **ROCK1P1** | **chr18** | **108128** | **C** | **A** | **heterozygosity** | **76** | **101** | **20** |
| **25** | **ncRNA_intronic** | **ROCK1P1** | **chr18** | **108131** | **T** | **C** | **heterozygosity** | **45** | **100** | **20** |
| **26** | **ncRNA_intronic** | **ROCK1P1** | **chr18** | **108165** | **G** | **T** | **heterozygosity** | **49** | **138** | **20** |
| **27** | **ncRNA_exonic** | **ROCK1P1** | **chr18** | **108309** | **T** | **A** | **homozygosity** | **91** | **130** | **20** |
| **28** | **ncRNA_exonic** | **ROCK1P1** | **chr18** | **108329** | **A** | **G** | **homozygosity** | **128** | **80** | **20** |
| **29** | **ncRNA_exonic** | **ROCK1P1** | **chr18** | **108344** | **G** | **C** | **homozygosity** | **115** | **44** | **20** |
| **30** | **ncRNA_splicing** | **ROCK1P1** | **chr18** | **108505** | **G** | **A** | **heterozygosity** | **107** | **85** | **20** |
| **31** | **ncRNA_intronic** | **ROCK1P1** | **chr18** | **108542** | **G** | **A** | **heterozygosity** | **3.01** | **58** | **20** |
| **32** | **ncRNA_intronic** | **ROCK1P1** | **chr18** | **108855** | **A** | **G** | **heterozygosity** | **18.1** | **38** | **20** |
| **33** | **ncRNA_intronic** | **ROCK1P1** | **chr18** | **108859** | **G** | **C** | **heterozygosity** | **39** | **38** | **20** |
| **34** | **intronic** | **ACAP2** | **chr3** | **1.97E+08** | **C** | **T** | **heterozygosity** | **61** | **35** | **20** |
| **35** | **intronic** | **TXNDC15** | **chr5** | **1.34E+08** | **A** | **G** | **homozygosity** | **170** | **37** | **20** |
| **36** | **intronic** | **TXNDC15** | **chr5** | **1.34E+08** | **A** | **G** | **homozygosity** | **115** | **39** | **20** |
| **37** | **intronic** | **TXNDC15** | **chr5** | **1.34E+08** | **C** | **T** | **homozygosity** | **108** | **41** | **20** |
| **38** | **intronic** | **TXNDC15** | **chr5** | **1.34E+08** | **A** | **G** | **homozygosity** | **147** | **34** | **20** |
| **39** | **intronic** | **TXNDC15** | **chr5** | **1.34E+08** | **G** | **A** | **homozygosity** | **132** | **34** | **20** |
| **40** | **intronic** | **TXNDC15** | **chr5** | **1.34E+08** | **G** | **A** | **homozygosity** | **64** | **66** | **20** |
| **41** | **intronic** | **TXNDC15** | **chr5** | **1.34E+08** | **C** | **T** | **homozygosity** | **89** | **83** | **20** |
| **42** | **intronic** | **TXNDC15** | **chr5** | **1.34E+08** | **G** | **A** | **homozygosity** | **133** | **80** | **20** |
| **43** | **intronic** | **TXNDC15** | **chr5** | **1.34E+08** | **C** | **T** | **homozygosity** | **155** | **73** | **20** |
| **44** | **intronic** | **TXNDC15** | **chr5** | **1.34E+08** | **T** | **C** | **homozygosity** | **154** | **73** | **20** |
| **45** | **intronic** | **TXNDC15** | **chr5** | **1.34E+08** | **A** | **G** | **homozygosity** | **164** | **127** | **20** |
| **46** | **intronic** | **TXNDC15** | **chr5** | **1.34E+08** | **G** | **A** | **homozygosity** | **185** | **134** | **20** |
| **47** | **intronic** | **TXNDC15** | **chr5** | **1.34E+08** | **T** | **C** | **homozygosity** | **190** | **121** | **20** |
| **48** | **intronic** | **TXNDC15** | **chr5** | **1.34E+08** | **T** | **C** | **homozygosity** | **197** | **117** | **20** |
| **49** | **intronic** | **TXNDC15** | **chr5** | **1.34E+08** | **G** | **A** | **homozygosity** | **188** | **118** | **20** |
| **50** | **intronic** | **TXNDC15** | **chr5** | **1.34E+08** | **A** | **G** | **homozygosity** | **190** | **120** | **20** |
| **51** | **intronic** | **TXNDC15** | **chr5** | **1.34E+08** | **G** | **A** | **homozygosity** | **186** | **108** | **20** |
| **52** | **intronic** | **TXNDC15** | **chr5** | **1.34E+08** | **C** | **T** | **homozygosity** | **182** | **109** | **20** |
| **53** | **intronic** | **TXNDC15** | **chr5** | **1.34E+08** | **G** | **A** | **homozygosity** | **202** | **95** | **20** |
| **54** | **intronic** | **TXNDC15** | **chr5** | **1.34E+08** | **G** | **T** | **homozygosity** | **198** | **89** | **20** |
| **55** | **intronic** | **TXNDC15** | **chr5** | **1.34E+08** | **T** | **C** | **homozygosity** | **193** | **79** | **20** |
| **56** | **intronic** | **TXNDC15** | **chr5** | **1.34E+08** | **C** | **T** | **homozygosity** | **197** | **77** | **20** |
| **57** | **intronic** | **TXNDC15** | **chr5** | **1.34E+08** | **G** | **A** | **homozygosity** | **190** | **63** | **20** |
| **58** | **intronic** | **TXNDC15** | **chr5** | **1.34E+08** | **C** | **T** | **homozygosity** | **176** | **62** | **20** |
| **59** | **intronic** | **TXNDC15** | **chr5** | **1.34E+08** | **A** | **T** | **homozygosity** | **169** | **45** | **20** |
| **60** | **intronic** | **TXNDC15** | **chr5** | **1.34E+08** | **A** | **G** | **homozygosity** | **134** | **34** | **20** |
| **61** | **intronic** | **TXNDC15** | **chr5** | **1.34E+08** | **C** | **A** | **homozygosity** | **133** | **35** | **20** |
| **62** | **intronic** | **TXNDC15** | **chr5** | **1.34E+08** | **G** | **A** | **homozygosity** | **127** | **44** | **20** |
| **63** | **intronic** | **TXNDC15** | **chr5** | **1.34E+08** | **G** | **A** | **homozygosity** | **153** | **54** | **20** |
| **64** | **intronic** | **TXNDC15** | **chr5** | **1.34E+08** | **A** | **G** | **homozygosity** | **150** | **67** | **20** |
| **65** | **intronic** | **TXNDC15** | **chr5** | **1.34E+08** | **G** | **A** | **homozygosity** | **156** | **96** | **20** |
| **66** | **intronic** | **TXNDC15** | **chr5** | **1.34E+08** | **A** | **G** | **homozygosity** | **153** | **104** | **20** |
| **67** | **intronic** | **TXNDC15** | **chr5** | **1.34E+08** | **A** | **G** | **homozygosity** | **159** | **101** | **20** |
| **68** | **intronic** | **TXNDC15** | **chr5** | **1.34E+08** | **C** | **G** | **homozygosity** | **160** | **56** | **20** |
| **69** | **intronic** | **TXNDC15** | **chr5** | **1.34E+08** | **C** | **T** | **homozygosity** | **170** | **44** | **20** |
| **70** | **intronic** | **TXNDC15** | **chr5** | **1.34E+08** | **T** | **C** | **homozygosity** | **164** | **36** | **20** |
| **71** | **intronic** | **TXNDC15** | **chr5** | **1.34E+08** | **T** | **C** | **homozygosity** | **168** | **94** | **20** |
| **72** | **intronic** | **TXNDC15** | **chr5** | **1.34E+08** | **T** | **C** | **homozygosity** | **187** | **135** | **20** |
| **73** | **intronic** | **TXNDC15** | **chr5** | **1.34E+08** | **T** | **C** | **homozygosity** | **180** | **110** | **20** |
| **74** | **intronic** | **TXNDC15** | **chr5** | **1.34E+08** | **A** | **G** | **homozygosity** | **169** | **131** | **20** |
| **75** | **intronic** | **TXNDC15** | **chr5** | **1.34E+08** | **T** | **C** | **homozygosity** | **162** | **129** | **20** |
| **76** | **intronic** | **TXNDC15** | **chr5** | **1.34E+08** | **G** | **A** | **homozygosity** | **137** | **126** | **20** |
| **77** | **intronic** | **TXNDC15** | **chr5** | **1.34E+08** | **G** | **A** | **homozygosity** | **173** | **136** | **20** |
| **78** | **intronic** | **TXNDC15** | **chr5** | **1.34E+08** | **A** | **G** | **homozygosity** | **175** | **106** | **20** |
| **79** | **intronic** | **TXNDC15** | **chr5** | **1.34E+08** | **G** | **A** | **homozygosity** | **188** | **113** | **20** |
| **80** | **intronic** | **TXNDC15** | **chr5** | **1.34E+08** | **A** | **G** | **homozygosity** | **190** | **114** | **20** |
| **81** | **intronic** | **TXNDC15** | **chr5** | **1.34E+08** | **C** | **T** | **homozygosity** | **199** | **141** | **20** |
| **82** | **intronic** | **TXNDC15** | **chr5** | **1.34E+08** | **G** | **A** | **homozygosity** | **199** | **145** | **20** |
| **83** | **intronic** | **TXNDC15** | **chr5** | **1.34E+08** | **C** | **T** | **homozygosity** | **201** | **118** | **20** |
| **84** | **intronic** | **TXNDC15** | **chr5** | **1.34E+08** | **C** | **T** | **homozygosity** | **196** | **103** | **20** |
| **85** | **intronic** | **TXNDC15** | **chr5** | **1.34E+08** | **A** | **G** | **homozygosity** | **200** | **101** | **20** |
| **86** | **intronic** | **TXNDC15** | **chr5** | **1.34E+08** | **A** | **G** | **homozygosity** | **203** | **76** | **20** |
| **87** | **intronic** | **TXNDC15** | **chr5** | **1.34E+08** | **A** | **G** | **homozygosity** | **168** | **60** | **20** |
| **88** | **exonic** | **TXNDC15** | **chr5** | **1.34E+08** | **T** | **C** | **homozygosity** | **169** | **33** | **20** |
| **89** | **exonic** | **TXNDC15** | **chr5** | **1.34E+08** | **C** | **A** | **homozygosity** | **160** | **43** | **20** |
| **90** | **exonic** | **TXNDC15** | **chr5** | **1.34E+08** | **C** | **T** | **homozygosity** | **159** | **42** | **20** |
| **91** | **exonic** | **TXNDC15** | **chr5** | **1.34E+08** | **A** | **G** | **homozygosity** | **154** | **37** | **20** |
| **92** | **exonic** | **TXNDC15** | **chr5** | **1.34E+08** | **G** | **A** | **homozygosity** | **132** | **38** | **20** |
| **93** | **exonic** | **TXNDC15** | **chr5** | **1.34E+08** | **G** | **A** | **homozygosity** | **133** | **43** | **20** |
| **94** | **exonic** | **TXNDC15** | **chr5** | **1.34E+08** | **G** | **A** | **homozygosity** | **130** | **64** | **20** |
| **95** | **UTR3** | **TXNDC15** | **chr5** | **1.34E+08** | **A** | **G** | **homozygosity** | **155** | **99** | **20** |
| **96** | **UTR3** | **TXNDC15** | **chr5** | **1.34E+08** | **T** | **C** | **homozygosity** | **157** | **82** | **20** |
| **97** | **UTR3** | **TXNDC15** | **chr5** | **1.34E+08** | **G** | **A** | **homozygosity** | **177** | **71** | **20** |
| **98** | **UTR3** | **TXNDC15** | **chr5** | **1.34E+08** | **A** | **G** | **homozygosity** | **189** | **64** | **20** |
| **99** | **UTR3** | **TXNDC15** | **chr5** | **1.34E+08** | **C** | **T** | **homozygosity** | **190** | **64** | **20** |
| **100** | **UTR3** | **TXNDC15** | **chr5** | **1.34E+08** | **T** | **C** | **homozygosity** | **192** | **60** | **20** |
| **101** | **UTR3** | **TXNDC15** | **chr5** | **1.34E+08** | **C** | **T** | **homozygosity** | **159** | **40** | **20** |
| **102** | **UTR3** | **TXNDC15** | **chr5** | **1.34E+08** | **A** | **G** | **homozygosity** | **137** | **40** | **20** |
| **103** | **UTR3** | **TXNDC15** | **chr5** | **1.34E+08** | **G** | **A** | **homozygosity** | **155** | **52** | **20** |
| **104** | **UTR3** | **TXNDC15** | **chr5** | **1.34E+08** | **A** | **G** | **homozygosity** | **162** | **54** | **20** |
| **105** | **UTR3** | **TXNDC15** | **chr5** | **1.34E+08** | **C** | **T** | **homozygosity** | **170** | **43** | **20** |
| **106** | **UTR3** | **TXNDC15** | **chr5** | **1.34E+08** | **G** | **A** | **homozygosity** | **172** | **47** | **20** |
| **107** | **UTR3** | **TXNDC15** | **chr5** | **1.34E+08** | **T** | **C** | **homozygosity** | **179** | **46** | **20** |
| **108** | **UTR3** | **TXNDC15** | **chr5** | **1.34E+08** | **T** | **C** | **homozygosity** | **180** | **45** | **20** |
| **109** | **UTR3** | **TXNDC15** | **chr5** | **1.34E+08** | **C** | **G** | **homozygosity** | **147** | **44** | **20** |
| **110** | **UTR3** | **TXNDC15** | **chr5** | **1.34E+08** | **T** | **G** | **homozygosity** | **176** | **41** | **20** |
| **111** | **UTR3** | **TXNDC15** | **chr5** | **1.34E+08** | **A** | **G** | **homozygosity** | **162** | **37** | **20** |
| **112** | **ncRNA_intronic** | **PGM5P2** | **chr9** | **68426275** | **C** | **A** | **heterozygosity** | **39** | **34** | **20** |
| **113** | **intronic** | **USP9Y** | **chrY** | **13464980** | **C** | **A** | **heterozygosity** | **16.1** | **32** | **20** |
