# Supplementary material for: Characterization of saliva microbiota’s functional feature based on metagenomic sequencing
Source: Springerplus. 2016 Dec 20;5(1):2098. doi: 10.1186/s40064-016-3728-6 (PMC5174016; doi:10.1186/s40064-016-3728-6)
Supplement: Supplementary file 2 — Additional file 2. SOM-supplementary online material [file 40064_2016_3728_MOESM2_ESM.doc]

**Supplementary Materials for the manuscript “Yang, Ning, et al, Preliminary characterization of saliva microbiota’s functional feature based on metagenomic sequencing”**

**Materials and Methods**

***Study design*** All human host volunteers (nearly 700 individuals) were from an oral health census on the undergraduates from the east campus of Sun Yat-sen University, Guangzhou, China, in September, 2009 [17]. Dental examinations were performed by five professional dentists who were previously trained and calibrated for the evaluation and sampling procedures, according to the criteria defined by the National Institute of Dental and Craniofacial Research criteria for caries diagnosis and recording (Kaste et al., 1996). The DMFT index measures the number of decayed, missing and filled teeth in epidemiologic surveys of dental caries (Anaise, 1984). It was adopted to measure each individual’s caries status and to define and distinguish the caries-sensitive subjects and healthy subjects. Finally, panoramic x-ray was applied to every volunteer to cross-validate the measured DMFT result. The intra-examiner reproducibility in both the pilot phase and the main survey was assessed by kappa statistics, which was higher than 0.91.

After oral health survey, “healthy” individuals (DMFT=0) and “caries-active(DMFT≧6) ” subjects (DMFT≧6) were chosen for saliva sample collection. All volunteers were given written informed consent in accordance with the sampling protocol with approval of the ethical committee of the Stomatology Hospital, Sun Yat-sen University. They were all unrelated individuals of both genders, aged between 18 and 23 years and shared a relatively homogeneous college-campus living environment. All reported no antibiotics intake for the preceding at least six months and no smoking or tobacco used. All were asked to avoid eating or drinking for 1h before oral sampling. Those with other oral (for example, periodontitis or halitosis) or systematic diseases were excluded. Subjects were comfortably seated and, after a few minutes of relaxation, they were trained to avoid swallowing saliva and asked to lean forward and spit all the saliva they produced for 5 minutes into a graduated test tube. Participants were asked to collect at least 5 ml unstimulated saliva in a plastic cup and sample were transferred to a plastic tube and stored at −80°C. To decipher the functional landscape of saliva microbiota, four saliva samples (including two from the “healthy” group and two from the “caries-active(DMFT≧6) ” group) were randomly selected for shotgun sequencing of total genomic DNA (**Table S1**).

***Whole-ecosystem sequencing of saliva*** For four of the saliva from the 46 samples (two from the healthy hosts and the other two from caries-active(DMFT≧6) hosts), shotgun pair-end libraries of total saliva genomic DNA were prepared respectively. Each metagenomic DNA libraries was then sequenced on one lane of pair-end 100bp or 75bp flow-cell on Solexa GA-IIx (Illumina, USA). Reads produced were processed via our computational pipelines customized for human oral microbiome analysis [16]. All sequences were deposited at Sequence Read Archive (http://www.ncbi.nih.gov/sra) under Accession ID SRA049721. The Solexa GA-IIx reads were subjected to quality filtering using FASTX-Toolkit (V0.0.10; http://hannonlab.cshl.edu/) which trimmed low quality read ends: the tailing nucleotides with quality less than 20 were removed. Human sequences were extracted after mapping the reads to human genome sequences using Novoalign (V2.06; http://www.novocraft.com); those paired reads with both mapping quality values exceeding 100 were labeled as host-originating reads and the rest were considered as microbiota-originating reads.

***Functional Classification of genes*** After removing the human-originating reads from each of the four whole-ecosystem sequencing datasets, over 8.4 million reads were present for each of the healthy saliva microbiota and over 33.1 million reads were for each of the caries-active(DMFT≧6) microbiota. Interestingly, the microbiota-originating reads constitute ~30% of the total number of reads for the two H samples, while they constitute ~50% of that for the two C samples (**Table S2**), suggesting the presence of a higher level of microbial DNA in the saliva of caries-active(DMFT≧6) hosts.

None-human short reads were assembled using SOAP [8]. Information for the raw reads and their assembly was presented in **Table S2.** Those contigs longer than 50bp were submitted to MG-RAST [2] for BLASTX against SEED database (http://www.theseed.org/). MG-RAST annotation was performed using an e-value cut-off of 1e-5 to identify abundant genes from the assembled reads [2,12]. About 7.1%~49.9% of the sequences contain predicted genes with known functions, while 24.6%~62.9% of the sequences encode predicted genes with unknown function (**Table S2**). Sequences were deposited, and publicly available on the MG-RAST server (H105: 4454806.3; H114-4454815.3; C201: 4454817.3; C218: 4454816.3). The functional assignment based on SEED subsystems was retrieved for four hierarchical levels: subsystem hierarchy 1, subsystem hierarchy 2, subsystem hierarchy 3 and function level. Functional assignment results based on SEED subsystems from additional metagenomic data in MG-RAST server were taken into comparison, including supragingival dental plaques from healthy individuals (MG-RAST IDs: NOCA_01P-4447192.3, NOCA_03P-4447102.3), supragingival dental plaques from caries-active(DMFT≧6) individuals (MG-RAST IDs: CA_04P-4447943.3, CA_06P-4447903.3), cavities plaques [1] (MG-RAST IDs: CA_06_1.6-4447971.3, CA_05_4.6-4447970.3), human gut microbiota [14] (MG-RAST IDs: TS1-4440452.7, TS2-4440453.6) and cow rumen microbiota (MG-RAST IDs: 640F6-4441679.3, 80F6-4441680.3). Heat maps and PCA (Principle Component Analysis) results of the functional gene composition were generated from MG-RAST using MEV v 2.0 [13] and R (version 2.9.1; <http://www.r-project.org/>).

MetaGene [10] was used to predict ORFs from the contigs. The predicted ORFs were aligned to each other using in-house Perl script to construct a non-redundant protein-coding gene set. These non-redundant protein sequences were aligned against the reference databases that include NR and KEGG (Kyoto Encyclopedia of Genes and Genomes; http://www.genome.jp/kegg/) using TBLASTN (e<10^-5^) and then analyzed by MEGAN [3]. The BLAST comparison thus allowed association of the proteins to their GO-term classification, as well as to a series of KO numbers. The genes and their associated information were thus projected based on their associated KO numbers onto the reference pathways in KEGG using the iPath tool [5].

***Genome and function linkage*** Human Microbiome Project (HMP) oral reference genomes were downloaded (<http://www.hmpdacc-resources.org/>) and concatenated into one large reference sequence. Alignment of the metagenomic reads to the HMP oral reference genomes was conducted using Bowtie [4] to access the abundance of these reference genomes in the four sequenced saliva microbiota (to define the dominant genomes) and the sequence coverage of these reference genomes or their close phylogenetic neighbors in the four microbiota. MG-RAST annotation based on SEED subsystem was retrieved and the extracted nucleotide sequences encoding proteins were searched against the HMP oral reference genomes using BLASTN to identity their originating genomes. The dominant functions in the four levels in SEED and dominant genomes were defined according to their relative abundance. A database recording the diversity, abundance and originating genomes of the genes and their functions was thus constructed based on which the relationship of the dominant function and the dominant genomes would be examined.

***Analysis of human-originating read***s The localization and distribution of Single-Nucleotide Polymorphisms (SNPs) on the four human-host genomes were obtained via the following strategy. *First*, alignments of the human reads to the reference human genome (version hg18) were performed using BWA [6]. *Second*, variants calling was performed using SAM tools (v 0.1.16) [7] and GATK (v1.0) [9] with default parameters, respectively. *Third*, the intersection of the variants predicted by SAM tools and GATK were taken as the final variant calls. Functional classification of point mutations was performed using ANNOVAR [15]. To test the correlation between the identified SNPs and the potential phenotypes of the human hosts, the SNPs were firstly filtered with the criteria of a minimal read depth of 30 (thus more likely to represent functional variations; [11]). Correlations of these filtered SNPs with potential diseases and phenotypes were subsequently identified by searching the Catalog of Published Genome-Wide Association Studies ([http://www.genome.gov/](http://www.genome.gov/page.cfm?pageid=26525384#download)).

1. Belda-Ferre P, Alcaraz LD, Cabrera-Rubio R, Romero H, Simon-Soro A, et al. (2012) The oral metagenome in health and disease. *ISME J* **6**: 46-56.

2. Glass EM, Wilkening J, Wilke A, Antonopoulos D, Meyer F. (2010) Using the metagenomics RAST server (MG-RAST) for analyzing shotgun metagenomes. *Cold Spring Harb Protoc* **2010**: pdb prot5368.

3. Huson DH, Auch AF, Qi J, Schuster SC. (2007) MEGAN analysis of metagenomic data. *Genome Res* **17**: 377-386.

4. Langmead B, Trapnell C, Pop M, Salzberg SL. (2009) Ultrafast and memory-efficient alignment of short DNA sequences to the human genome. *Genome Biol* **10**: R25.

5. Letunic I, Yamada T, Kanehisa M, Bork P. (2008) iPath: interactive exploration of biochemical pathways and networks. *Trends Biochem Sci* **33**: 101-103.

6. Li H, Durbin R. (2009) Fast and accurate short read alignment with Burrows-Wheeler transform. *Bioinformatics* **25**: 1754-1760.

7. Li H, Handsaker B, Wysoker A, Fennell T, Ruan J, et al. (2009) The Sequence Alignment/Map format and SAMtools. *Bioinformatics* **25**: 2078-2079.

8. Li R, Yu C, Li Y, Lam TW, Yiu SM, et al. (2009) SOAP2: an improved ultrafast tool for short read alignment. *Bioinformatics* **25**: 1966-1967.

9. McKenna A, Hanna M, Banks E, Sivachenko A, Cibulskis K, et al. (2010) The Genome Analysis Toolkit: a MapReduce framework for analyzing next-generation DNA sequencing data. *Genome Res* **20**: 1297-1303.

10. Noguchi H, Park J, Takagi T. (2006) MetaGene: prokaryotic gene finding from environmental genome shotgun sequences. *Nucleic Acids Res* **34**: 5623-5630.

11. Oetting WS. (2011) Exploring the functional consequences of genomic variation: the 2010 Human Genome Variation Society Scientific Meeting. *Hum Mutat* **32**: 486-490.

12. Overbeek R, Begley T, Butler RM, Choudhuri JV, Chuang HY, et al. (2005) The subsystems approach to genome annotation and its use in the project to annotate 1000 genomes. *Nucleic Acids Res* **33**: 5691-5702.

13. Saeed AI, Bhagabati NK, Braisted JC, Liang W, Sharov V, et al. (2006) TM4 microarray software suite. *Methods Enzymol* **411**: 134-193.

14. Turnbaugh PJ, Hamady M, Yatsunenko T, Cantarel BL, Duncan A, et al. (2009) A core gut microbiome in obese and lean twins. *Nature* **457**: 480-484.

15. Wang K, Li M, Hakonarson H. (2010) ANNOVAR: functional annotation of genetic variants from high-throughput sequencing data. *Nucleic Acids Res* **38**: e164.

16. Xie G, Chain PS, Lo CC, Liu KL, Gans J, et al. (2010) Community and gene composition of a human dental plaque microbiota obtained by metagenomic sequencing. *Mol Oral Microbiol* **25**: 391-405.

17. Yang F, Zeng X, Ning K, Liu KL, Lo CC, et al. (2012) Saliva microbiomes distinguish caries-active from healthy human populations. *ISME J* **6**: 1-10.
